# Abundant diversity of accessory genetic elements and associated antimicrobial resistance genes in *pseudomonas aeruginosa* isolates from a single Chinese hospital

**DOI:** 10.1186/s12941-023-00600-3

**Published:** 2023-06-29

**Authors:** Xiaofei Mu, Xinyue Li, Zhe Yin, Ying Jing, Fangzhou Chen, Huixia Gao, Zhi Zhang, Yueyang Tian, Huiqian Guo, Xiuhui Lu, Jiaqi He, Yali Zheng, Dongsheng Zhou, Peng Wang, Erhei Dai

**Affiliations:** 1 Department of Clinical Laboratory Medicine, Hebei Medical University, Shijiazhuang, Hebei 050011 China; 2 Department of Laboratory Medicine, the Fifth Hospital of Shijiazhuang, Hebei Medical University, No. 42 Tanan Road, Yuhua District, Shijiazhuang, Heibei 050021 China; 3 State Key Laboratory of Pathogen and Biosecurity, Beijing Institute of Microbiology and Epidemiology, No. 20, Dongdajie, Fengtai, Beijing, 100071 China

**Keywords:** *Pseudomonas aeruginosa*, Genome sequencing, MLST, T3SS-related virulotypes, Accessory genetic elements, Antimicrobial resistance

## Abstract

**Objectives:**

*Pseudomonas aeruginosa* has intrinsic antibiotic resistance and the strong ability to acquire additional resistance genes. However, a limited number of investigations provide detailed modular structure dissection and evolutionary analysis of accessory genetic elements (AGEs) and associated resistance genes (ARGs) in *P. aeruginosa* isolates. The objective of this study is to reveal the prevalence and transmission characteristics of ARGs by epidemiological investigation and bioinformatics analysis of AGEs of *P. aeruginosa* isolates taken from a Chinese hospital.

**Methods:**

Draft-genome sequencing was conducted for *P. aeruginosa* clinical isolates (n = 48) collected from a single Chinese hospital between 2019 and 2021. The clones of *P. aeruginosa* isolates, type 3 secretion system (T3SS)-related virulotypes, and the resistance spectrum were identified using multilocus sequence typing (MLST), polymerase chain reaction (PCR), and antimicrobial susceptibility tests. In addition, 17 of the 48 isolates were fully sequenced. An extensive modular structure dissection and genetic comparison was applied to AGEs of the 17 sequenced *P. aeruginosa* isolates.

**Results:**

From the draft-genome sequencing, 13 STs were identified, showing high genetic diversity. BLAST search and PCR detection of T3SS genes (*exoT*, *exoY*, *exoS*, and *exoU*) revealed that the *exoS*+*/exoU*- virulotype dominated. At least 69 kinds of acquired ARGs, involved in resistance to 10 different categories of antimicrobials, were identified in the 48 *P. aeruginosa* isolates. Detailed genetic dissection and sequence comparisons were applied to 25 AGEs from the 17 isolates, together with five additional prototype AGEs from GenBank. These 30 AGEs were classified into five groups -- integrative and conjugative elements (ICEs), unit transposons, Inc_pPBL16_ plasmids, Inc_p60512−IMP_ plasmids, and Inc_pPA7790_ plasmids.

**Conclusion:**

This study provides a broad-scale and deeper genomics understanding of *P. aeruginosa* isolates taken from a single Chinese hospital. The isolates collected are characterized by high genetic diversity, high virulence, and multiple drug resistance. The AGEs in *P. aeruginosa* chromosomes and plasmids, as important genetic platforms for the spread of ARGs, contribute to enhancing the adaptability of *P. aeruginosa* in hospital settings.

**Supplementary Information:**

The online version contains supplementary material available at 10.1186/s12941-023-00600-3.

## Background

*Pseudomonas aeruginosa* is a common nosocomial pathogen [1] responsible for approximately 10% of all nosocomial infections [2]. Its extensive virulence and strong ability to evade antimicrobial therapeutic activity makes *P. aeruginosa* one of the most dangerous bacterial pathogens and is a major reason for its morbidity and mortality in immunocompromised individuals [3].

*P. aeruginosa* has a non-clonal epidemic population structure with 1392 sequence types (STs) defined worldwide (last accessed 5 May, 2022). The top 10 *P. aeruginosa* high-risk clones (ST235, ST111, ST233, ST244, ST357, ST308, ST175, ST277, ST654, and ST298) [4] are widespread in hospitals [5–7]. The success of *P. aeruginosa* high-risk clones likely reflects the acquisition and accumulation of antimicrobial resistance genes (ARGs) carried on accessory genetic elements (AGEs), given the large number s of distinct ARGs and AGEs detected in *P. aeruginosa* high-risk clones. AGEs, such as integrative and conjugative elements (ICEs), plasmids, and integrons, play a critical role in the accumulation and spread of ARGs in *P. aeruginosa* [10–15]. ICEs encode self-integration and self-conjugation modules, typically composed of an *attL* (attachment site at the left end), *int* (integrase), *xis* (excisionase), *rlx* (relaxase), *oriT* (origin of conjugative replication), *cpl* (coupling protein), a P (TivB)- or F (TivF)- type T4SS machinery (mating pair formation), and an *attR* (attachment site at the right end). Plasmid-mediated transmission of ARGs among *P. aeruginosa* has been widely reported, such as the IncP-2 plasmid carrying *bla*_IMP−45_ [16], the IncP-6 plasmid carrying *bla*_KPC−2_ [17], and the IncU plasmid carrying *bla*_KPC−2_ [18]. These plasmids harbor diverse resistance genes that can be horizontally transmitted in hospital settings. AGEs are often inserted within the genomes of *P. aeruginosa* by site-specific recombination events, facilitating the rapid spread of ARGs within communities and hospitals. Genetic analyses of AGEs from in-hospital *P. aeruginosa* contribute to our understanding of the transmission and infection dynamics of highly resistant bacteria isolates [7]. While many reports exist on AGEs and their associated ARGs among *P. aeruginosa*, few provide detailed modular structure dissection and evolutionary analysis of resistance genes in *P. aeruginosa* isolates from a single hospital.

In this study, we collected 48 *P. aeruginosa* isolates from a single Chinese hospital and conducted draft-genome sequencing to analyze the prevalence of STs and T3SS virulotypes. Moreover, we fully sequenced 17 of the 48 isolates to measure the prevalence of ARG-associated AGEs. From these fully sequenced isolates, we genetically dissected the modular structures of 25 AGEs and performed a detailed sequence comparison of these AGEs together with five prototype AGEs from GenBank. Data presented here provided a deeper understanding of the bioinformatics and epidemiology of ARGs and AGEs in *P. aeruginosa* from a single hospital.

## Methods

### Bacterial strains and identification

A total of 48 *P. aeruginosa* isolates (Table S2) were recovered from patients with nosocomial infections in a Chinese public hospital from 2019 to 2021. Bacterial antimicrobial susceptibility was tested by VITEK 2 Compact (BioMerieux, NC, USA), and interpreted as per the 2020 Clinical and Laboratory Standards Institute (CLSI) guidelines [19].

### PCR identification

Primers of the *exoT*, *exoY*, *exoS*, and *exoU* genes were designed (data not shown, https://www.ncbi.nlm.nih.gov/tools/primer-blast). PCR was performed in a 30 µl volume using a ProFlex PCR System (Applied Biosystems, CA, USA). The reaction mixture (30 µl) consisted of 15 µl 2x Taq PCR Master Mix (MT201, Biomed, China), 0.3 µl each forward and reverse primers, 3 µl template DNA, and 11.4 µl ddH_2_O. The reaction mixture with no template DNA was included as a negative control. The cycling programs consisted of 1 × 94 °C for 3 min, 30 × 94 °C for 40 s, 60 °C (*exoT*, *exoY*, and *exoU*), 55°C (*exoS*) for 40 s and 72 °C for 1 min, and 1 × 72 °C for 5 min. After completion of all cycles, the PCR products were examined in 1.5% agarose (TSJ001, Tsingke, China) gel electrophoresis in the presence of TS-gelred (TSJ002, Tsingke, China) (7 µl/100ml gel). The stained gels were then visualized under UV light and photographed using iBringht 750 (Thermo Fisher, MA, USA).

### Sequencing, and sequence assembly

All these 48 isolates were subjected to draft-genome sequencing using a paired-end library with an average insert size of 350 bp (range 150–600 bp) on a HiSeq sequencer (Illumina, CA, USA). In addition, 17 of them were subjected to complete-genome sequencing with a sheared DNA library with an average size of 15 kb (range 10–20 kb) on a PacBio RSII sequencer (Pacific Biosciences, CA, USA) (Table S1). The quality control analysis of sequencing data was conducted using NanoPack [20] and FastQC (https://www.bioinformatics.babraham.ac.uk/projects/fastqc). Sequence assembly and annotation were performed as described previously [21, 22].

### Sequence annotation and comparison

Open reading frames (ORFs) and pseudogenes were predicted using RAST 2.0 [23] combined with BLASTP/BLASTN searches [24] against the UniProtKB/Swiss-Prot database [25] and the RefSeq database [26]. Annotation of resistance genes, AGEs, and other genome features was carried out using online databases including CARD [27], ResFinder [28], ISfinder [29], INTEGRALL [30], and Tn Number Registry [31]. Multiple and pairwise sequence comparisons were performed using MUSCLE 3.8.31 [32] and BLASTN, respectively. Gene organization diagrams were drawn in Inkscape 1.0 (https://inkscape.org/en/). Heatmaps were plotted with MeV 4.9.0 [33].

### Multi-Locus sequence typing

The sequence types (STs) of *P. aeruginosa* isolates were identified according to the online *P. aeruginosa* MLST scheme (https://pubmlst.org/paeruginosa/). New STs discovered in our study were submitted to the curator of the database. GoeBURST was used for the MLST analysis, demonstrating the allelic relationship and prevalence of various STs. In this study, isolates were classified into the same clonal complex (CC) if six of the seven alleles were homologous. The PHYLOViZ 2.0 platform was used for data management and analysis of CCs, which were defined by related ST clusters exhibiting variation in a single locus (single locus variants-SLV) or in two loci (double locus variants-DLV) [34].

### Conjugal transfer

Conjugal transfer experiments were carried out with rifampin-resistant *P. aeruginosa* ATCC 27,853 as a recipient, and the wild-type *P. aeruginosa* isolate as a donor. Three milliliters of overnight cultures of each of donor and recipient bacteria was mixed together, harvested, and resuspended in 80 mL of Brain Heart Infusion (BHI) broth (BD Biosciences). The mixture was spotted on a 1 cm^2^ hydrophilic nylon membrane filter with a 0.45 μm pore size (Millipore) that was placed on BHI agar (BD Biosciences) plate and then incubated for mating at 37 °C for 12–18 h. Bacteria were then washed from the filter membrane and spotted on Muller–Hinton (MH) agar (BD Biosciences) plates to select *aadB*-carrying ATCC 27,853 transconjugants. Transconjugant selection was done using 1500 mg/mL rifampin (for ATCC 27,853) together with 200 mg/L gentamicin (for *aadB*).

## Results

### Sample source and antimicrobial resistance profile of 48 clinical isolates

**Fig. 1:**
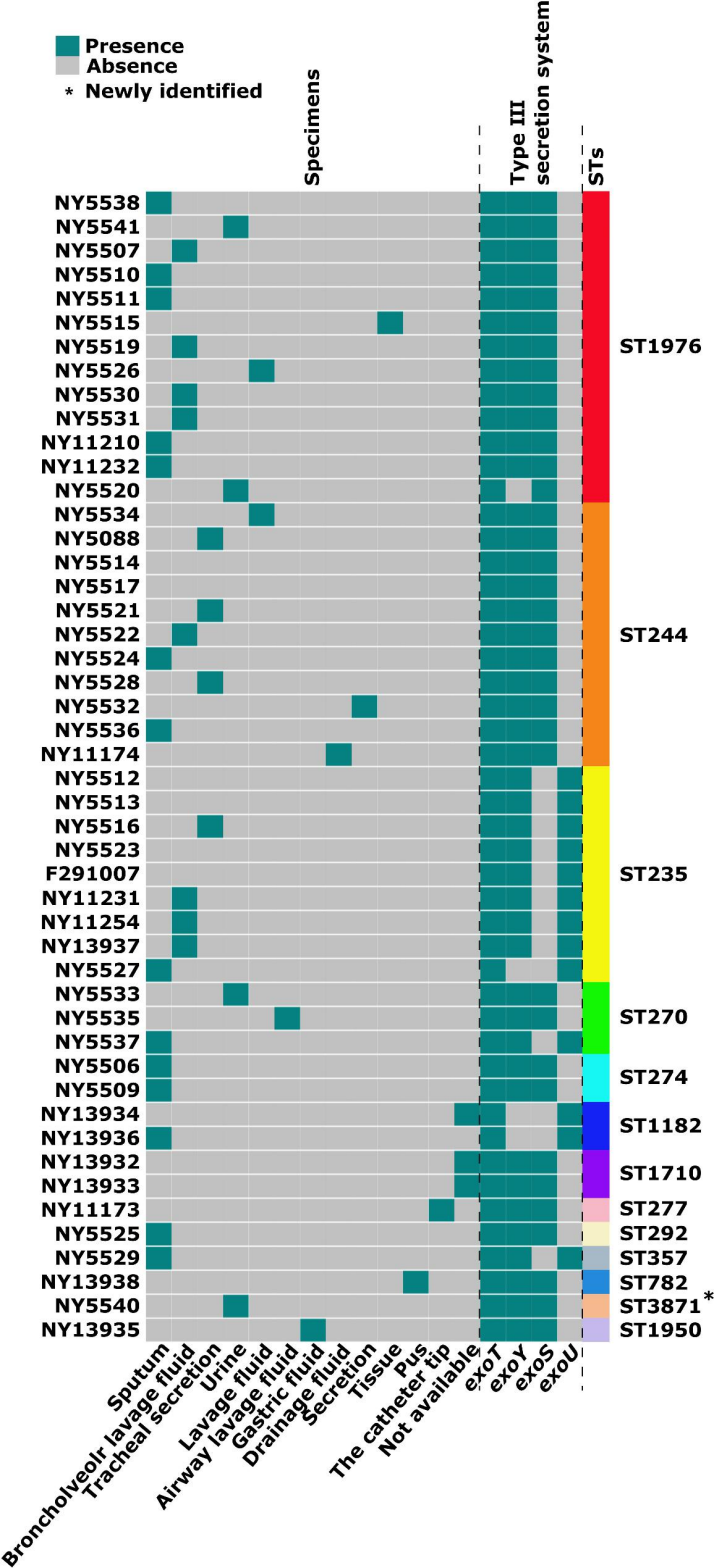
Heatmap of specimens, virulence genes, and STs of the 48 isolates

We collected 48 *P. aeruginosa* isolates from a hospital in the Henan Province of China. These isolates were mostly recovered from lung infection samples, such as sputum, bronchoalveolar lavage fluid, tracheal secretion, and airway lavage fluid (Fig. 1). Antimicrobial susceptibility/resistance profiles of these 48 isolates were described using nine different antimicrobials (Table S1). A high prevalence of multi-drug resistance (MDR) was observed, with 91.67% (n = 44) of isolates resistant to three classes of antibiotics.

### Identification of 13 STs, T3SS virulotypes, and acquired ARGs from the 48 clinical ***P. aeruginosa*** isolates

We performed multi-locus sequence typing (MLST) analysis on 48 *P. aeruginosa* isolates and identified 13 STs presented in descending order by the number of isolates in which the ST was found: ST1976 (n = 13), ST244 (n = 11), ST235 (n = 9), ST270 (n = 3), ST274 (n = 2), ST1182 (n = 2), ST1710 (n = 2), ST277 (n = 1), ST292 (n = 1), ST357 (n = 1), ST782 (n = 1), ST3871 (n = 1), and ST1950 (n = 1) (Fig. 1 and Table S1).

ST3871 is a novel ST of *P. aeruginosa*. ST244, ST235, ST277, and ST357 are included in the worldwide top 10 *P. aeruginosa* high-risk clones [4] and were present in 45.8% of the 48 isolates. Thus, the *P. aeruginosa* isolates collected had high genetic diversity and many high-risk clones were prevalent in the hospital.

Based on BLAST search and PCR for the *exoT*, *exoY*, *exoS*, and *exoU* genes, we found *exoT*+/*exoY*+/*exoS*+/*exoU-* (34/48, 70.8%) was the predominant T3SS virulotype, (Fig. 1 and Table S1). The *exoT* and *exoY* genes were common in *P. aeruginosa*. All isolates that lacked the *exoU* gene carried the *exoS* gene. The widespread presence and diverse virulotypes of the T3SS in clinical isolates of *P. aeruginosa* suggest that the bacterial clones circulating and spreading were mostly those with high virulence.

At least 69 acquired ARGs involved in resistance to 10 different categories of antimicrobials were identified in the 48 *P. aeruginosa* isolates (Fig. S2). β-lactam-resistance genes, aminoglycoside-resistance genes, phenicol-resistance genes, and sulfonamide-resistance genes were distributed across all isolates.

### Collection of 30 AGEs for sequence comparison

**Fig. 2:**
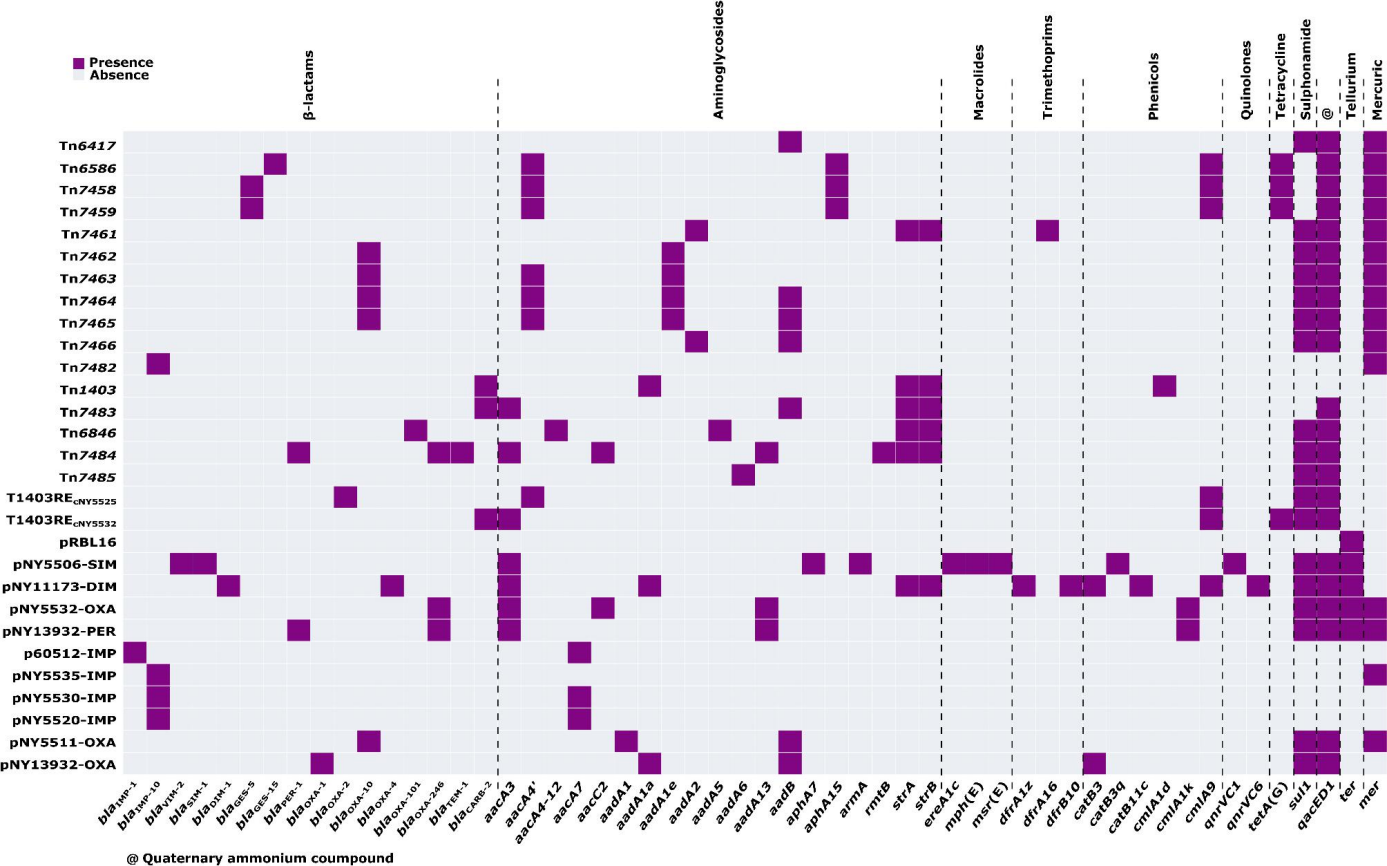
Heatmap of prevalence of resistance genes in the 30 AGEs. All data are provided in Table S2

The 25 AGEs sequenced in this study and five additional reference/prototype AGEs (Tn*6417*, Tn*1403*, pRBL16, p60512-IMP, and pPA7790) from GenBank were compared in a detailed sequence comparison (Table 1). The 30 AGEs were classified into five groups: (i) integrative and conjugative elements (ICEs), (ii) unit transposons, (iii) Inc_pPBL16_ plasmids, (iv) Inc_p60512−IMP_ plasmids, and (v) Inc_pPA7790_ plasmids. Each group is discussed in detail below. At least 54 ARGs, involved in resistance to 11 different categories of antimicrobials and heavy metals were identified in these 30 AGEs (Fig. 2 and Table S2).

**Table 1:** Major features of the 30 AGEs characterized in this study

Isolate	Chromosome or plasmid			Transposon or AGE contained		Integron contained	Reference
	Designation	Accession number	Classification	Designation	Classification		
DHS01	cDHS01	CP013993	–	Tn*6417*	Tn*6417*-related ICEs	In159	[1]
NY11254	cNY11254	CP096960	–	Tn*6586*		In1820	This study
F291007	cF291007	CP081345	–	Tn7458*		In717	
NY5523	cNY5523	CP096941	–	Tn7459*		In717 and In2144*	
NY5532	cNY5532	CP096950	–	Tn7461*		In36	
NY5530	cNY5530	CP096946	–	Tn7462*		In1815*	
NY5507	cNY5507	CP096929	–	Tn7463*		In2044*	
NY5520	cNY5520	CP096937	–	Tn7464*		In1836*	
NY5510	cNY5510	CP096932	–	Tn7465*		In1818*	
NY11210	cNY11210	CP096958	–	Tn7466*		In1979*	
NY5511	cNY5511	CP096934	–	Tn7482*		In995	
RPL11	pRPL11	AF313472	–	Tn*1403*	Tn*1403*-related transposons or derivatives	In28	[2]
NY13936	cNY13936	CP096964	–	Tn7483*		In1791	This study
NY5524	cNY5524	CP096942	–	Tn*6846*		In1079	
NY5525	cNY5525	CP096945	–	Tn7484*		In458	
NY5523	cNY5523	CP096941	–	Tn7485*		In51	
NY5525	cNY5525	CP096945	–	T1403RE_cNY5525_		In1829*	
NY5532	cNY5532	CP096950	–	T1403RE_cNY5532_		In44 and In167	
SJTE-3	pRBL16	CP015879	Inc_pRBL16_	–	–	–	[3]
NY5506	pNY5506-SIM	CP096928		T1403RE_pNY5506 − SIM_	Tn*1403*-related derivatives	In56, In1809* and In1810*	This study
NY11173	pNY11173-DIM	CP096957		T1403RE_pNY11173 − DIM_		In1980*, In1225 and In1226	
NY5532	pNY5532-OXA	CP096951		T1403RE_pNY5532 − OXA_		In1281	
NY13932	pNY13932-PER	CP096963		T1403RE_pNY13932 − PER_		In1281	
60,512	p60512-IMP	MF344578	Inc_p60512−IMP_^&^	Tn*6394*	IS*Pa17*-based transposition units	In992	[4]
NY5535	pNY5535-IMP	CP096955		Tn7486*		In995	This study
NY5530	pNY5530-IMP	CP096949		Tn7487*		In1814*	
NY5520	pNY5520-IMP	CP096938		Tn7488*		In1835*	
NY5511	pNY5511-OXA	CP096935		Tn7494*		In1819*	
PA7790	pPA7790	CP015000	Inc_pPA7790_^&^	–	–	–	[5]
NY13932	pNY13932-OXA	CP096962		11.54-kb MDR region	–	In2151*	This study

^*^, Newly identified; ^&^, newly designated

### Comparison of 11 Tn***6417***-related ICEs

Tn*6417* (108.2 kb in length) was used as the reference ICE [35]. It was initially described in *P. aeruginosa* DHS01 [36]. The backbones of Tn*6586*, Tn*7458*, Tn*7459*, Tn*7461*, Tn*6417*, Tn*7462*, Tn*7463*, Tn*7464*, Tn*7465*, Tn*7466*, and Tn*7482* varied in size from approximately 71.5 kb to nearly 95.1 kb, but all contained *attL/R*, *int*, *cpl* (coupling protein), *rlx* (relaxase), and an F-type T4SS gene set (Fig. S3). In addition, their backbones had at least three major modular differences: (i) the presence of a unique *xerC*–to–*orf1068* region only in Tn*6417*; (ii) the presence of *orf672*, *orf306*, *piL1*–to–*orf381*, and *orf3336*–to–*orf2514* regions in Tn*7462*, Tn*7463*, Tn*7464*, Tn*7465*, Tn*7466*, and Tn*7482*; and (iii) *orf384*–to–*orf765* and *orf1419*–to–*rlx* regions from Tn*7462*, Tn*7463*, Tn*7464*, Tn*7465*, Tn*7466*, and Tn*7482* displayed < 90% nucleotide identity with their counterparts in Tn*6586*, Tn*7458*, Tn*7459*, Tn*7461*, and Tn*6417*.

Each of the 11 ICEs carried a single accessory module, as follows for ICE (accessory module): Tn*6586* (Tn*6809*), Tn*7458* (Tn*7404*), Tn*7459* (Tn*7405*), Tn*7461* (Tn*7460*), Tn*6417* (Tn*6532*), Tn*7462* (In1815), Tn*7463* (In2044), Tn*7464* (In1836), Tn*7465* (In1818), Tn*7466* (In1979), and Tn*7482* (In995) (Fig. S3).

**Fig. 3:**
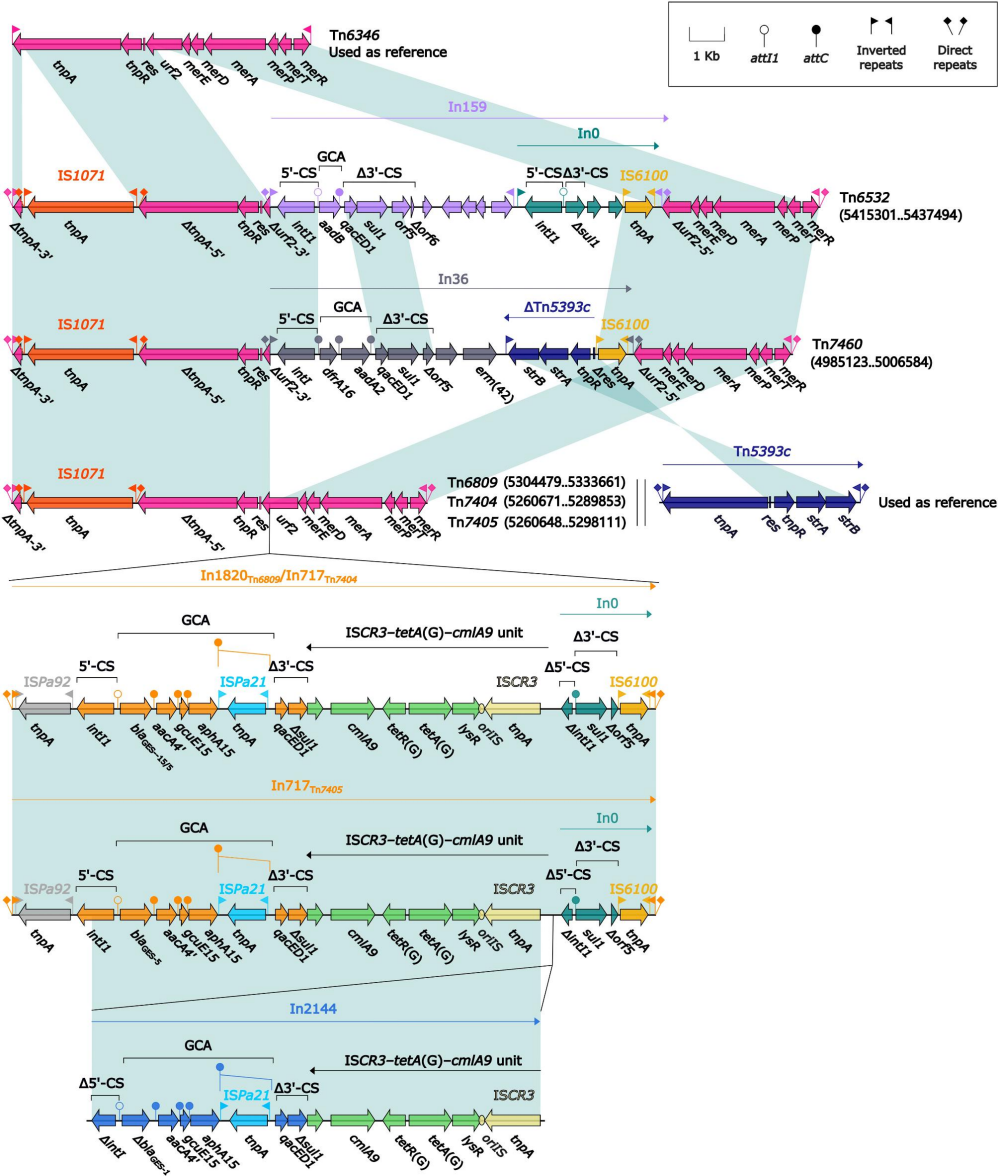
Comparison of six Tn*6346*-related transposons. Genes are denoted by arrows. Genes, AGEs, and other features are colored based on their functional classification. Shading denotes regions of homology (nucleotide identity ≥ 95%). Numbers in brackets indicate nucleotide positions within the chromosomes of strains DHS01, NY5532, NY11254, F291007, and NY5523, respectively. The accession number of Tn*6346* used as reference is EU696790.

In the first five ICEs, transposons were integrated at a site upstream of the ICE backbone gene *orf582* and identified as derivatives of Tn*6346*. Tn*6346*, a prototype Tn*3*-family unit transposon originally identified in *Achromobacter* spp. AO22 [37], manifested as a hybrid of the core transposition module *tnpAR–res* from Tn*5051* and the *mer* region from Tn*501* (Fig. 3). The five Tn*6346* derivatives differed from Tn*6346* in two major aspects: (i) insertion of IS*1071* at the same position within *tnpA* in all five Tn*6346* derivatives and (ii) insertion of five different concise class 1 integrons (In159, In36, In1820, In717_Tn*7404*_, and In717_Tn*7405*_) into *urf2*.

In159 harbored a gene cassette array (GCA) with *aadB* as its only gene. In36 had the GCA *dfrA16*–*aadA2*. In1820, In717_Tn*7404*_, and In2044 in In717_Tn*7405*_ had the GCAs *bla*_GES_–*aacA4’*–*gcuE15*–*aphA15* with different *bla*_GES_ subtypes: *bla*_GES−15_, *bla*_GES−5_, and *bla*_GES−1_, respectively. (Fig. 3).

**Fig. 4:**
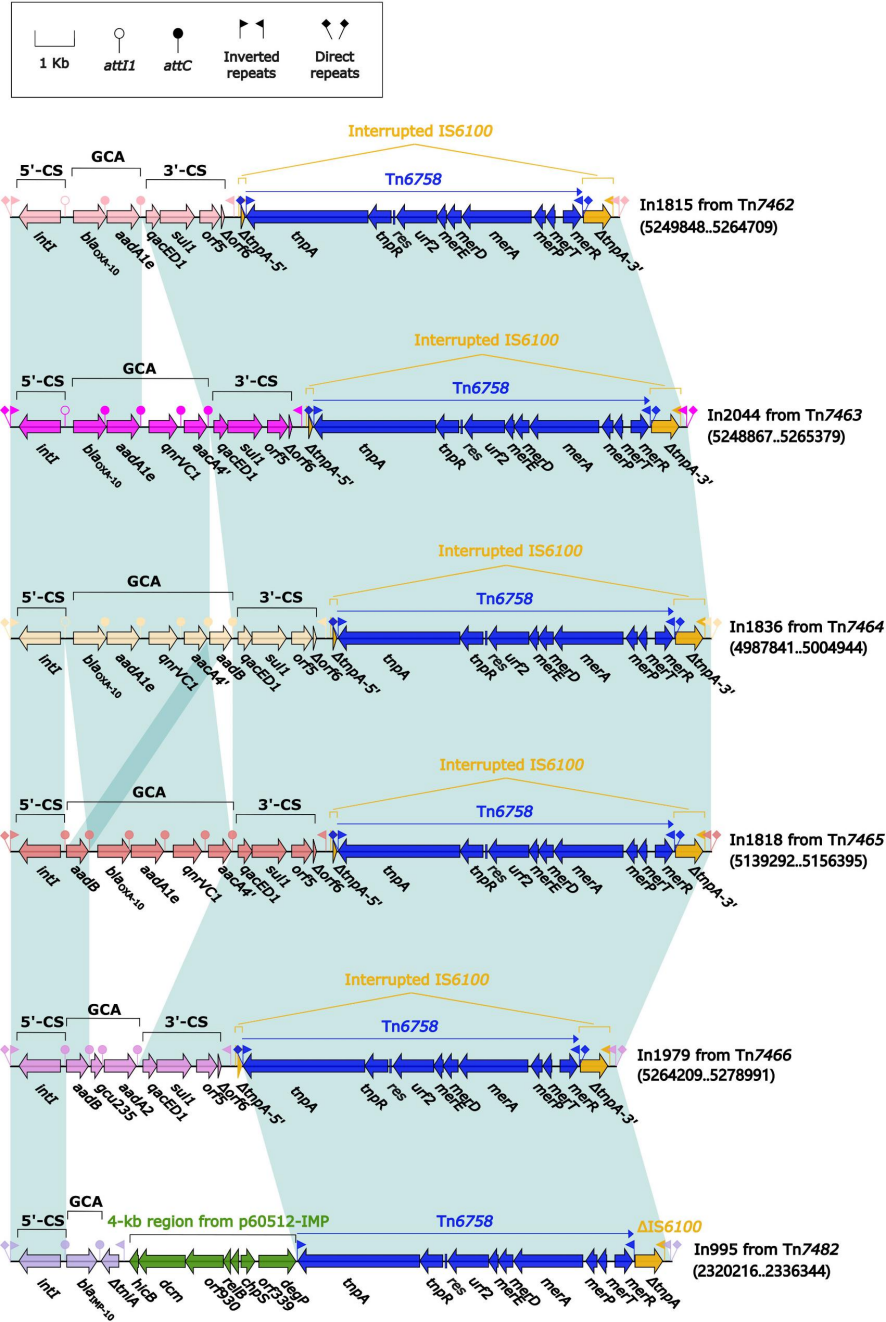
Organization of In1815, In2044, In1836, In1818, In1979, and In995. Genes are denoted by arrows. Genes, AGEs, and other features are colored based on their functional classification. Shading denotes regions of homology (nucleotide identity ≥ 95%). Numbers in brackets indicate nucleotide positions within the chromosomes of strains NY5530, NY5507, NY5520, NY5510, NY11210, and NY5511, respectively

Each of the remaining six Tn*6417*-related ICEs had integrons integrated, including In1815, In2044, In1836, In1818, In1979, and In995, respectively (Fig. 4).These six integrons had the same insertion:Tn*6758*, a prototype Tn*3*-family unit transposon initially found in *Achromobacter xylosoxidans* X02736 [38]. Five of the Tn*6758* interrupted the *tnpA* of IS*6100*, but in Tn*7482*, the insertion of Tn*6758* plus a 4.0-kb region from p60512-IMP resulted in the truncation of IS*6100*.

The GCAs *bla*_OXA−10_–*aadA1e*, *bla*_OXA−10_–*aadA1e*–*qnrVC1*–*aacA4´*, *bla*_OXA−10_–*aadA1e*–*qnrVC1*–*aacA4´*–*aadB* in In1815, In2044, and In1836 were incremental. This phenomenon demonstrates the process of accumulation and evolution of ARGs through integrons. In addition, the organization of In1836 and In1818 were highly consistent, with one difference in the position of *aadB.* In1979 had the GCA *aadB*–*aadA2*. In995 had the shortest GCA, which contained only *bla*_IMP−10_ (Fig. 4).

### Comparison of seven Tn***1403***-related elements

**Fig. 5:**
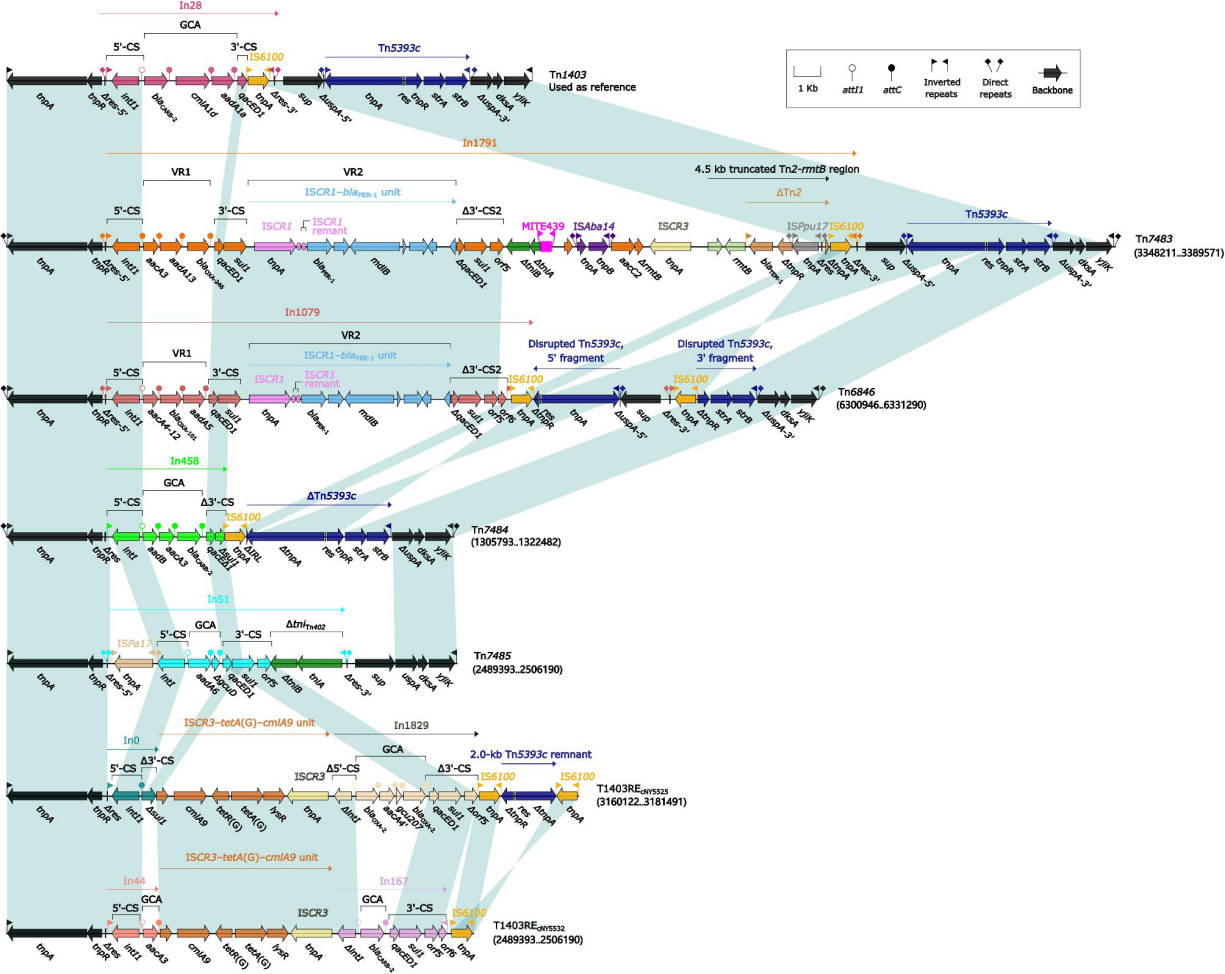
Comparison of Tn*1403* and six related elements. Genes are denoted by arrows. Genes, AGEs, and other features are colored based on their functional classification. Shading denotes regions of homology (nucleotide identity ≥ 95%). Numbers in brackets indicate nucleotide positions within the chromosomes of strains NY13936, NY5524, NY5525, NY5523, NY5525, and NY5532, respectively. The accession number of Tn*1403* used as reference is AF313472.

Tn*1403*, a Tn*3*-family prototype unit transposon, was originally found in *P. aeruginosa* plasmid RPL11 [39] and displayed a backbone structure *IRL–tnpAR–res–sup–uspA–dksA–yjiK–IRR*, with the integration of two accessory modules In28 and Tn*5393c* into *res* and *uspA*, respectively [40]. We identified six chromosome-borne Tn*1403* derivatives: Tn*7483*, Tn*6846*, Tn*7484*, Tn*7485*, T1403RE_cNY5525_, and T1403RE_cNY5532_ (Fig. 5). None of these T1403RE elements could be recognized as intact transposons due to the truncation of relevant core transposition modules. These six Tn*1403* derivatives differed from Tn*1403* in three major aspects: (i) integration of different class 1 integrons In1791, In1079, In458, In51, In1829, In44, and In167, instead of In28; (ii) absence of Tn*5393c* in Tn*7485* and T1403RE_cNY5532_; and (iii) insertion of the IS*CR3–tetA*(G)*–cmlA9* unit into T1403RE_cNY5525_ and T1403RE_cNY5532_.

In1791 and In1079, two complex class 1 integrons, differed in variable region 1 (VR1) but were the same in VR2. The five concise class1 integrons In458, In51, In1829, In44, and In167 exhibited three additional major modular differences: (i) insertion of IS*Pa17* only in In51; (ii) the presence of different GCAs; and (iii) insertion of Δ*tni*_Tn*402*_ only in In51.

### Comparison of five Inc_pRBL16_ plasmids

Inc_pRBL16_ was originally identified and designated in *P. citronellolis* plasmid pRBL16 [41]. pRBL16 represented the Inc_pRBL16_ reference plasmid as it was the complete Inc_pRBL16_ backbone without any exogenous insertions. The four plasmids pNY5506-SIM, pNY11173-DIM, pNY5532-OXA, and pNY13932-PER were assigned to the Incp_RBL16_ group (Table 1), because each harbored a *repA* (replication initiation) gene sharing > 96% nucleotide identity to *repA*_IncpRBL16_ and contained a conserved Inc_pRBL16_ backbone (> 98% nucleotide identity with > 90% coverage and highly similar gene organization).

The Inc_pRBL16_ backbone genes or gene loci *repA*_IncpRBL16_ together with its iterons (replication), *parB2–parAB* (partition), *cpl* and several *tivF* genes (conjugal transfer), *che* (chemotaxis), *pil* (pilus assembly) and *ter* (tellurium resistance), were conserved among all five plasmids. Moreover, 2–20 kb deletions of backbone regions occurred in the four plasmids because of the insertion of exogenous genetic material.

**Fig. 6:**
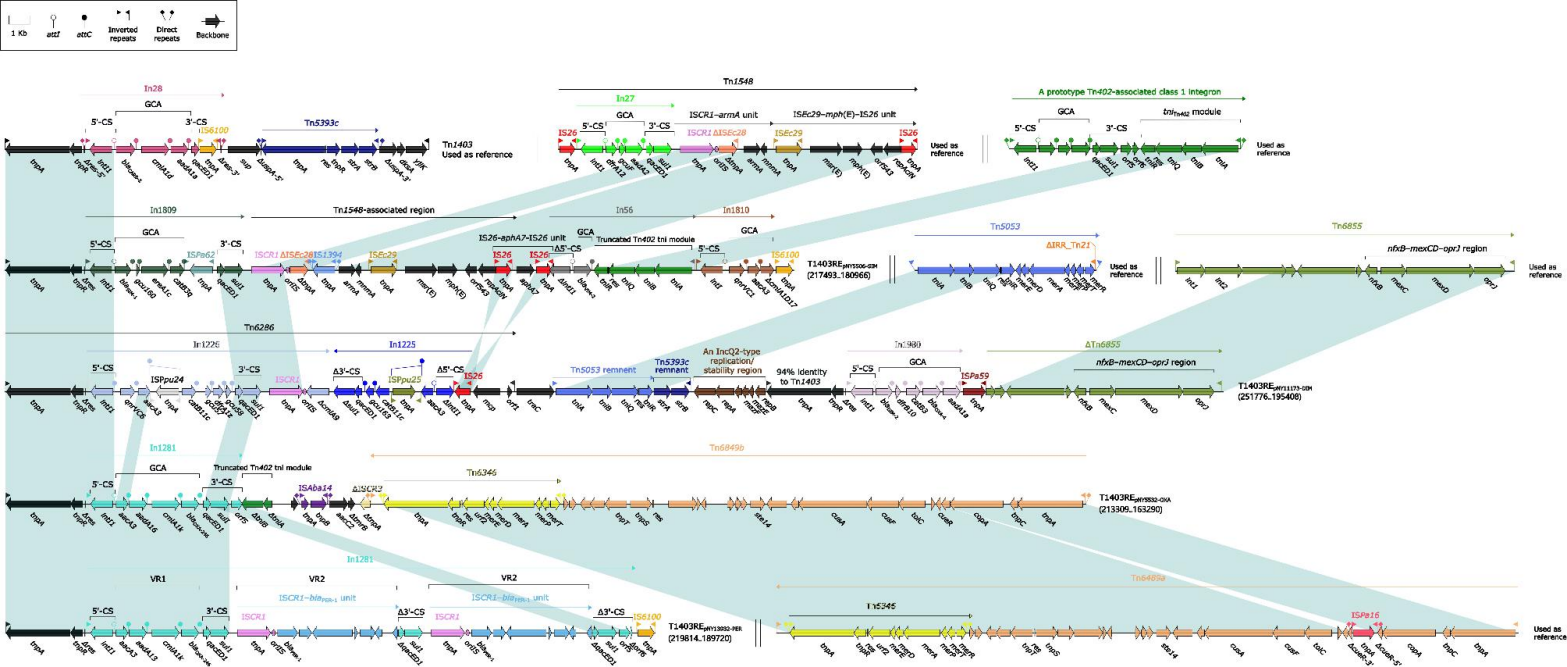
Comparison of Tn*1403* and its four related derivatives. Genes are denoted by arrows. Genes, AGEs, and other features are colored based on their functional classification. Shading denotes regions of homology (nucleotide identity ≥ 95%). The numbers in brackets indicate nucleotide positions within the plasmids of strains NY5506, NY11173, NY5532, and NY13932 respectively. The accession numbers of Tn*1403*, Tn*1548*, Tn*5053*, Tn*6855*, and Tn*6489a* used as reference are AF313472, AF550415, L40585, MK347425, and CP017969, respectively

Compared to pRBL16, each of the other four Inc_pRBL16_ plasmids acquired 3–5 accessory modules integrated at various sites across the Inc_pRBL16_ backbone (Fig. S4). Four transposon-like elements carried 5´-terminal regions (IRL*–tnpAR–Δres*) of Tn*1403* and were named T1403RE_pNY5506 − SIM_, T1403RE_pNY11173 − DIM_, T1403RE_pNY5532 − OXA_, and T1403RE_pNY13932 − PER_. These elements displayed considerable modular diversification including: (i) the presence of diverse class 1 integrons with different collections of resistance genes; and (ii) the presence of various transposons with different collections of resistance genes (Fig. 6).

### Comparison of five Inc_p60512−IMP_ plasmids

Four plasmids (pNY5535-IMP, pNY5530-IMP, pNY5520-IMP, and pNY5511-OXA) could not be assigned to any known Inc groups. A novel Inc_p60512−IMP_ group is thus proposed. p60512-IMP was used as the Inc_p60512−IMP_ reference plasmid. It was originally identified and described in *P. aeruginosa* 60,512 in our previous studies [42]. The four plasmids pNY5535-IMP, pNY5530-IMP, pNY5520-IMP, and pNY5511-OXA harbored not only homologous *repA* genes (100% nucleotide identity to *repA*_Incp60512−IMP_) together with its iterons but also similar backbone gene organization with p60512-IMP (> 99% nucleotide identity with > 98% coverage).

Seven accessory regions were inserted at three different sites within the backbones of the five Inc_p60512−IMP_ plasmids: (i) Tn*7486*, Tn*7487*, Tn*7488*, Tn*6394*, and Tn*7494* plus adjacent Tn*6758* were inserted at the site upstream of *pine* (DNA specific recombinase) in pNY5535-IMP, pNY5530-IMP, pNY5520-IMP, p60512-IMP, and pNY5511-OXA, respectively; (ii) Tn*6758* was inserted into *orf222* in pNY5535-IMP; and (iii) Tn*5403* was inserted at the site upstream of Δ*orf498* in pNY5520-IMP (Fig. S5).

**Fig. 7:**
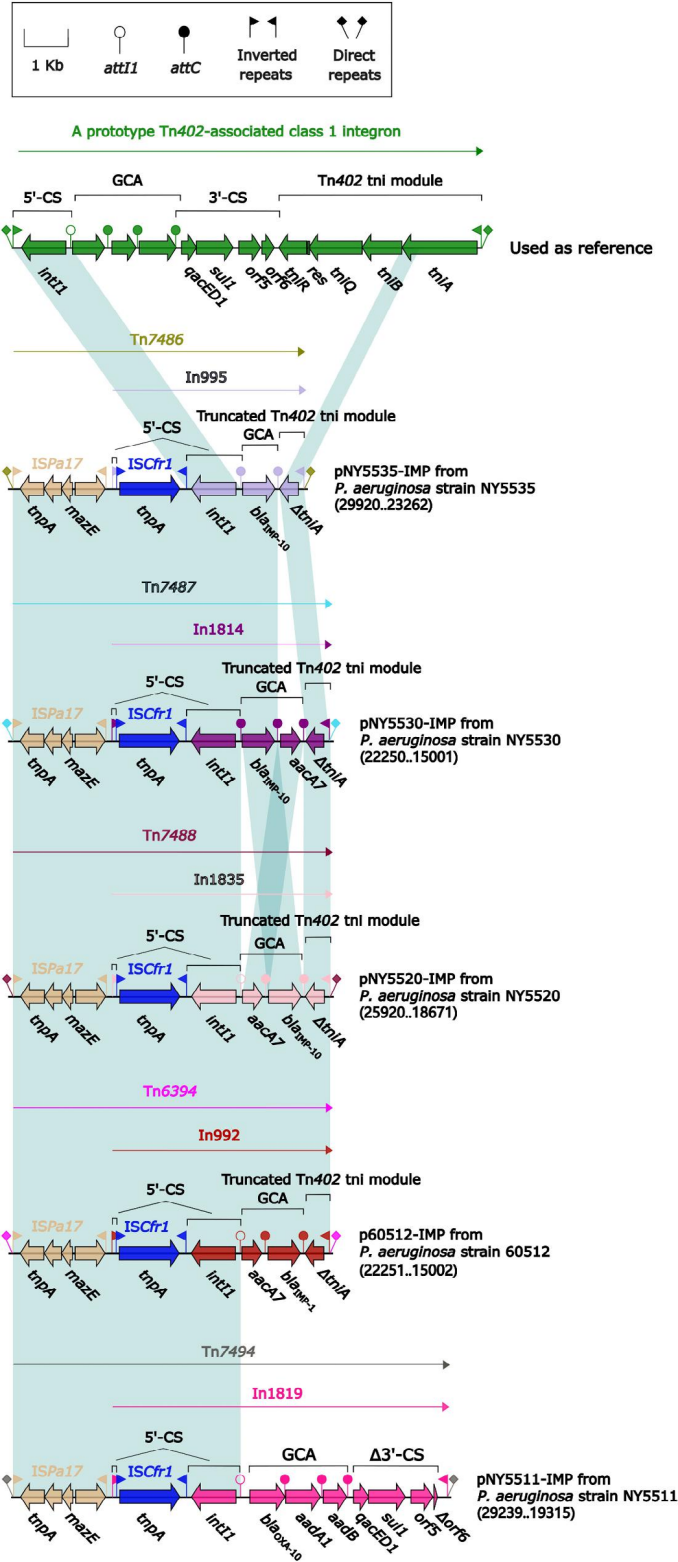
Comparison of five IS*Pa17*-based transposition units Tn*6394*, Tn*7486*. Tn*7487*, Tn*7488*, and Tn*7494*. Genes are denoted by arrows. Genes, AGEs, and other features are colored based on their functional classification. Shading denotes regions of homology (nucleotide identity ≥ 95%). Numbers in brackets indicate nucleotide positions within the plasmids of strains NY5535, NY5530, NY5520, and NY5511, respectively. The accession number of Tn*6394* used as reference is MF344578

Tn*7486*, Tn*7487*, Tn*7488*, Tn*6394*, and Tn*7494* were composed of an IS*Pa17* element (encoding a transposase, a recombinase, and a toxin/anti-toxin system) [43] together with In995, In1814, In1835, In992, or In1819, respectively, at its downstream end (Fig. 7). The connection of IS*Pa17* with each of these integrons might create the high levels of nucleotide identity between the IRL/IRR of IS*Pa17* and the IRi/IRt of these Tn*402*-associated class 1 integrons. In995, In1814, In1835, In992, and In1819 harbored GCAs *bla*_IMP−10_, *bla*_IMP−10_–*aacA7*, *aacA7*–*bla*_IMP−10_, *aacA7*–*bla*_IMP−1_, and *bla*_OXA−10_–*aadA1*–*aadB*–*qacED1*, respectively. The truncated *tni* modules of In995, In1814, In1835, and In992 are expected to impair the mobility of their respective plasmids.

### Comparison of two Inc_pPA7790_plasmids

Plasmid pNY13932-OXA could not be assigned to any known Inc groups. A novel Inc_pPA7790_ group was thus designated. Plasmid pPA7790, initially extracted from *P. aeruginosa* PA7790 [44], was used as the reference because it was the first sequenced plasmid carrying *repA*_IncpPA7790_ without any exogenous insertions. pNY13932-OXA was assigned to Inc_pPA7790_ due to the *repA* sharing > 98% nucleotide identity to *repA*_IncpPA7790_ and conserved Inc_pPA7790_ backbones (> 96% nucleotide identity with > 93% coverage; highly similar gene organization).

A comparative genomics analysis was applied to the two Inc_pPA7790_ plasmids. The Inc_pPA7790_ backbone genes included *repAB*_IncpPA7790_ together with its iterons (replication), *parAB* (partition), *rlx*–*traM*–*tivB*–*pilL* genes (conjugal transfer), *topA* (DNA topoisomerase), *radC* (DNA repair protein), and *ssb* (single-stranded DNA-binding protein) (Fig. S6).

**Fig. 8:**
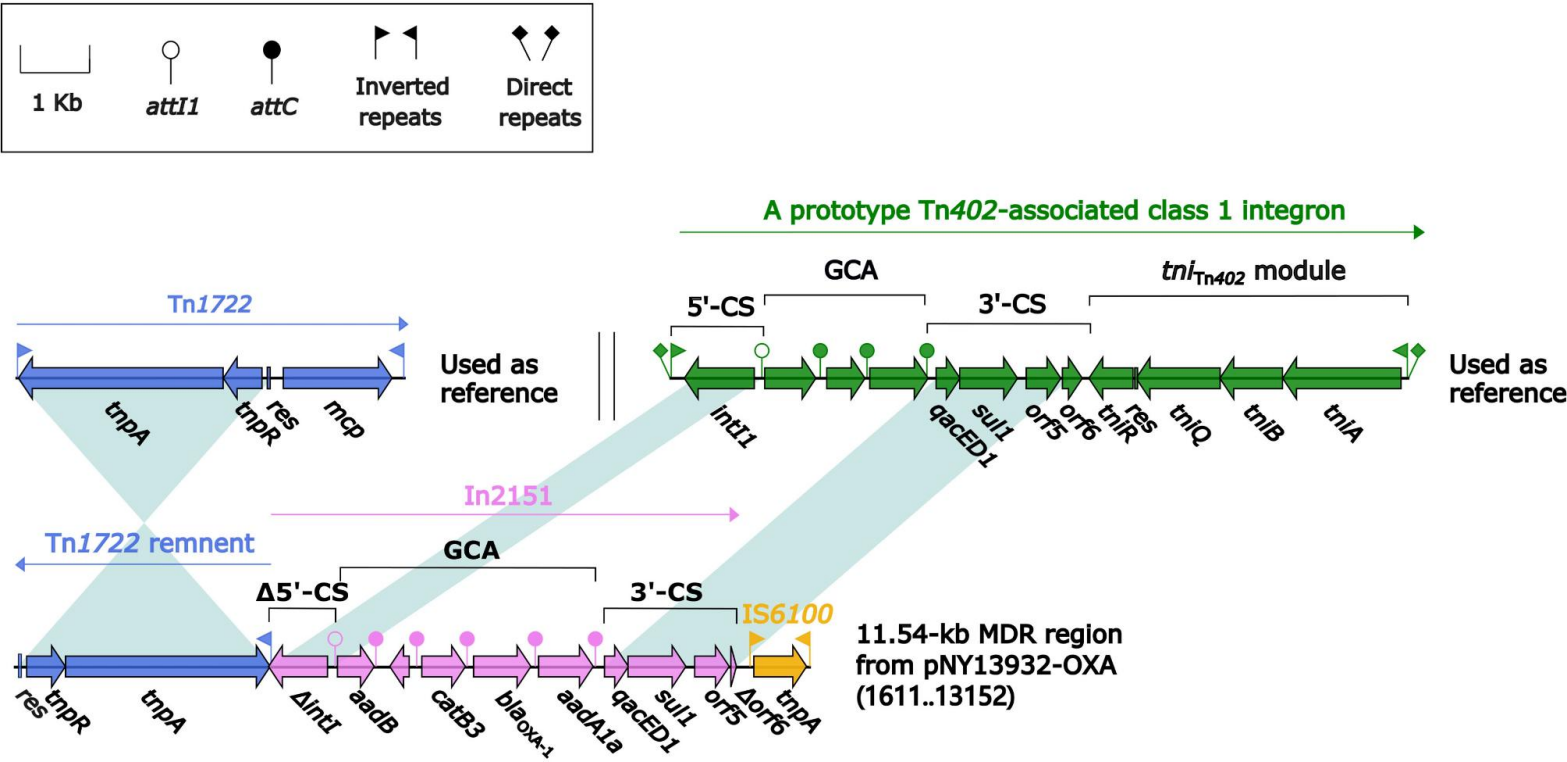
Organization of 11.54-kb MDR region in pNY13932-OXA. Genes are denoted by arrows. Genes, AGEs, and other features are colored based on their functional classification. Shading denotes regions of homology (nucleotide identity ≥ 95%). Numbers in brackets indicate nucleotide positions within the plasmids of strains NY13932. The accession number of Tn*1722* used as reference is X61367

Compared with pPA7790, an 8.7-kb deletion in pNY13932-OXA was replaced by an 11.54-kb MDR region inserted at the site downstream of *orf285*. It was composed of a 3.7-kb Tn*1722* remnant (*IRL*–*tnpAR*–*res*) plus a concise class 1 integron In2151 carrying GCA *aadB*–*catB3*–*bla*_OXA−1_–*aadA1a*, and one IS*6100*. The *bla*_OXA−1_, *catB3*, *aadB*, and *aadA1a* genes mediate resistance to β-lactam, phenicol, and aminoglycosides (Fig. 8).

### Summary of newly identified or designated AGEs

We identified 33 new AGEs. Of these, 16 were directly integrated into chromosomes or plasmids, including nine ICEs: Tn*7458*, Tn*7459*, Tn*7461*, Tn*7462*, Tn*7463*, Tn*7464*, Tn*7465*, T*n7466*, and Tn*7482*; four IS-based transposition units: Tn*7486*, Tn*7487*, Tn*7488*, and Tn*7494*; three unit transposons: Tn*7483*, Tn*7484*, and Tn*7485*. The remaining 17 AGEs were inner components of the above 16 and included three unit transposons (Tn*7404*, Tn*7405*, Tn*7460*) and 14 integrons (In2144, In1815, In2044, In1836, In1818, In1979, In1829, In1809, In1810, In1980, In1814, In1835, In1819, and In2151). Moreover, two novel Inc groups Inc_p60512−IMP_ and Inc_pPA7790_ were designated in this study.

**Transferability and antimicrobial susceptibility.** pNY13932-OXA, selected to represent Inc_pPA7790_, transferred from the wild-type isolate NY13932 into ATCC 27,853 through conjugation, generating the transconjugant ATCC 27,853/pNY13932-OXA. Both NY13932 and ATCC 27,853/pNY13932-OXA showed high resistance to gentamicin with a minimum inhibitory concentration (MIC) value ≥ 128 µg/mL owing to the production of aminoglycoside-2’-adenylyltransferase.

## Discussion

The *P. aeruginosa* isolates collected are genetically diverse and belong to mostly high-risk clones, including ST235, ST244, ST277, and ST357. All ST235 isolates contained the *exoU* gene, as has been found in previous studies, which leads to poor clinical outcomes and early mortality [45, 46]. All ST244 isolates contained the *exoS* gene, which is also consistent with previous literature [4, 47, 48]. These observations provide evidence that clonal lineages are linked to specific T3SS virulotypes, and this linkage may play a major role in their intrinsic virulence levels.

In addition, all 13 *exoU* + isolates are resistant to β-lactams and 12 are resistant to fluoroquinolones. Nearly all (97.1%; 34/35,) *exoS* + isolates are resistant to β-lactams. Many studies have reported that high virulence isolates often also show multidrug resistance, especially fluoroquinolone resistance [49–51]. This correlation between T3SS virulotypes and antimicrobial resistance suggests a coevolutionary response leading to the formation of high-risk clones [8]. Thus, the identification of virulence genes facilitates the prediction of resistance patterns and guides antibiotic treatment.

Among the nine ST235 isolates in this study, class A β-lactamases, including multiple GES and PER variants, were detected most frequently. This observation is consistent with previously reported results [9, 52, 53]. OXA variants are the most widespread β-lactamases among the ST244 isolates of the current study, followed by PER-1 and TEM-116, which matches previous data indicating ST244 is associated with multiple different β-lactamases [54, 55]. The ST277 and ST357 isolates encode DIM and OXA enzymes, respectively. This suggests that a widely successful clone has more opportunities to encounter and acquire ARGs, which may explain the high prevalence and diversity of ARGs in *P. aeruginosa* high-risk clones.

The combination of T3SS-related virulence and antimicrobial resistance facilitates the emergence and spread of high-risk clones in hospitals to some extent, leading *P. aeruginosa* to become a leading cause of morbidity and mortality in cystic fibrosis patients and immunocompromised individuals. Our study illustrates the emerging trends and threats of *P. aeruginosa* high-risk clones in a hospital setting in the era of antimicrobial resistance.

Of the 54 ARGs found in the 30 AGEs in the current study, 49 are found in class 1 integrons. These integrons, which have captured various ARGs, are further integrated into ICEs and plasmids with intercellular mobility and relevant unit transposons with intracellular mobility. The remaining five ARGs were found in other various subregions: the gene *aphA7* in the IS*26*-*aphA7*-IS*26* unit, the genes *armA*, *mph*(E), and *msr*(E) in the Tn*1548* related region and *ter* in the Inc_pRBL16_ plasmid backbone region. Therefore, dissecting the genome structure of *P. aeruginosa* reveals that ARGs are always dependent on a variety of vectors for expression and spread.

Two groups of AGEs provide intuitive insight into the evolution of integrons: (i) the gradual accumulation of GCAs observed in In1815, In2044, In1836, and In1818 from Tn*6417*-related ICEs that show up as *bla*_OXA−10_–*aadA1e*, *bla*_OXA−10_–*aadA1e*–*qnrVC1*–*aacA4´*, *bla*_OXA−10_–*aadA1e*–*qnrVC1*–*aacA4´*–*aadB*, and *aadB*–*bla*_OXA−10_–*aadA1e*–*qnrVC1*–*aacA4´* (Fig. 5); and (ii) the accumulation, transversion, and substitution of GCAs seen in In995, In1814, In1835, and In992 from Inc_p60512−IMP_ plasmids show up as GCAs *bla*_IMP−10_, *bla*_IMP−10_–*aacA7*, *aacA7*–*bla*_IMP−10_, and *aacA7*–*bla*_IMP−1_,respectively (Fig. 7). GCA sharing can generate diversity within members of a bacterial clone or species [56, 57], and a diverse collection of GCAs plays an important role in enabling *P. aeruginosa* to overcome the deleterious effects of antibiotics [58] and provides an added survival advantage in specific or challenging environments, such as hospitals.

Up to 150 subregions were identified in the 30 AGEs in this study, including 32 unit transposons, 39 integrons, 11 putative resistance units, 66 insertion sequences (ISs), a Tn*1548* associated region, and a 4.5-kb truncated Tn*2*-*rmtB* region. These intact or residue subregions together constitute the diverse mosaic structure of AGEs that are created by complex transposition and homologous recombination, facilitate horizontal genetic exchange and promote the acquisition and spread of resistance genes.

Our conjugal transfer experiment confirms the Inc_pPA7790_ plasmid pNY13932-OXA can to transfer between cells. Furthermore, the horizontal transferability of Tn*6417*-related ICEs and Inc_p60512−IMP_ plasmids was demonstrated in our previous studies [42, 59, 60]. Of note, Inc_p60512−IMP_ plasmids are transferred through electroporation rather than conjugation because they contain only four conjugal transfer genes (*traA*, *traC*, *traD* and *cpl*), which is insufficient for plasmid conjugation [43, 61].

All identified AGEs were initially described in *P. aeruginosa*, except the Inc_pRBL16_ plasmid, which was originally identified in *P. citronellolis*. Tn*6417*-related ICEs have spread worldwide in numerous bacterial species, including *P. aeruginosa* [59, 60, 62], and, less frequently, in *Klebsiella pneumoniae* (accession number CP085740), *Bordetella* (accession number CP049957), *Alcaligenes* (accession number CP032153), *Achromobacter xylosoxidans* (accession number LN890477), *Morganella morganii* (accession number CP061513), *Aeromonas caviae* (accession number AP024402), *Casimicrobium huifangae* (accession number CP041352), and *Delftia acidovorans* (accession number CP000884). Tn*1403*-related regions have a wide range of hosts and are frequently identified in Pseudomonadaceae [40, 63–65], including *P. putida* (accession number CP045551), *P. asiatica* (accession number CP101701), *P. juntendi* (accession number CP091088), *P. soli* (accession number CP009365), *P. fulva* (accession number CP064945), *P. migulae* (accession number CP043572), *P. shirazica* (accession number CP063457), and also sporadically in *Aeromonas media* (accession number CP047963). The Inc_p60512−IMP_ plasmids have been most often identified in *P. aeruginosa* [42, 66] and occasionally in *Achromobacter* (accession number KM659090). By contrast, the Inc_pRBL16_ plasmids and the Inc_pPA7790_ plasmids have been identified only in *P. aeruginosa* to date (last accessed 9 September, 2022). In conclusion, the high prevalence of these five groups of AGEs in *Pseudomonas* isolates is surprising, suggesting that they might already be widespread in Chinese hospital and play a dominant role in the spread of ARGs.

## Conclusion

The five groups of AGEs display a high-level diversification in modular structures, have complex mosaic natures, and carry many ARGs. Integration of these ARG-containing AGEs into *P. aeruginosa* likely contributes to the accumulation and dissemination of ARGs in *P. aeruginosa*, enhancing the adaptability of *P. aeruginosa* in hospital settings and its survivability under drug selection pressure. To better understand the prevalence of STs, T3SS virulotypes, and AGEs in hospital settings and to assess the clinical implications for humans, a comprehensive survey of *P. aeruginosa* is necessary.

## Electronic supplementary material

Below is the link to the electronic supplementary material.

Supplementary Material 1: Figure S1. A minimum spanning tree of MLST among *P. aeruginosa isolates.* This analysis is displayed by the goeBURST diagram. The MLST database (http://pubmlst.org/paeruginosa, last accessed May 5th, 2022) contained 8251 isolates producing a total of 3912 sequence types (STs). A cluster of linked isolates corresponds to a CC (clonal complex). 2635 STs are divided into 492 CCs and 1277 STs The red boxes show the STs of the 48 isolates in this study. Blue rots are the others in the *P. aeruginosa* MLST database.

Supplementary Material 2: Figure S2. Heatmap of prevalence of resistance genes in the 48 clinical isolates.

Supplementary Material 3: Figure S3. Comparison of 11 Tn*6417-*>related ICEs. Genes are denoted by arrows. Genes, AGEs, and other features are colored based on their functional classification. Shading in light blue denotes regions of homology (nucleotide identity ≥ 95%), light orange (nucleotide identity < 90%). The accession number of Tn*6417* used as reference is EU696790.

Supplementary Material 4: Figure S4. Comparison of five Inc_pRBL16_ plasmids pRBL16, pNY5506-SIM, pNY11173-DIM, pNY5532-OXA, and pNY13932-PER. Genes are denoted by arrows. Genes, AGEs, and other features are colored based on their functional classification. Shading denotes regions of homology (nucleotide identity ≥ 95%). The accession number of pRBL16 used as reference is CP015879.

Supplementary Material 5: Figure S5. Comparison of five Inc_p60512−IMP_ plasmids p60512-IMP, pNY5535-IMP, pNY5530-IMP, pNY5520-IMP, and pNY5511-OXA. Genes are denoted by arrows. Genes, AGEs, and other features are colored based on their functional classification. Shading denotes regions of homology (nucleotide identity ≥ 95%). The accession number of p60512-IMP used as reference is MF344578.

Supplementary Material 6: Figure S6. Comparison of two Inc_pPA7790_ plasmids pPA7790 and pNY13932-OXA. Genes are denoted by arrows. Genes, AGEs, and other features are colored based on their functional classification. Shading denotes regions of homology (nucleotide identity ≥ 95%). The accession number of pPA7790 used as reference is CP015000.

Supplementary Material 7

Supplementary Material 8

## Data Availability

The complete chromosome sequences of NY5507,NY5510, NY5511, NY5530, NY11210, NY5520, NY5524, NY5532, NY5523, F291007, NY11254, NY5535, NY5506, NY13936, NY13932, NY11173, and NY5525 and those plasmids of pNY5507-IMP, pNY5511-OXA, pNY5530-IMP, pNY5520-IMP, pNY5520-KPC, pNY5532-OXA, pNY5535-IMP, pNY5506-SIM, pNY13932-OXA, pNY13932-PER, and pNY11173-DIM were submitted to GenBank under accession numbers CP096929, CP096932, CP096934, CP096946, CP096958, CP096937, CP096942, CP096950, CP096941, CP081345, CP096960, CP096953, CP096927, CP096964, CP096961, CP096956, CP096945, CP096930, CP096935, CP096949, CP096938, CP096939, CP096951, CP096955, CP096928, CP096962, CP096963, and CP096957, respectively.

## Data Availability

The data used and analyzed during this study are available from the corresponding author on reasonable request.

## Abbreviations

ST: Sequence type
T3SS: Type III secretion system
ARG: Antimicrobial resistance gene
AGE: Accessory genetic element
ICE: Integrative and conjugative element
GCA: Gene cassette array
MDR: Multi-drug resistance
MLST: Multi-locus sequence typing
CLSI: Clinical and Laboratory Standards Institute
