# Supplementary figures and images for: Abundant diversity of accessory genetic elements and associated antimicrobial resistance genes in pseudomonas aeruginosa isolates from a single Chinese hospital
Source: Ann Clin Microbiol Antimicrob. 2023 Jun 29;22:51. doi: 10.1186/s12941-023-00600-3 (PMC10311859; doi:10.1186/s12941-023-00600-3)

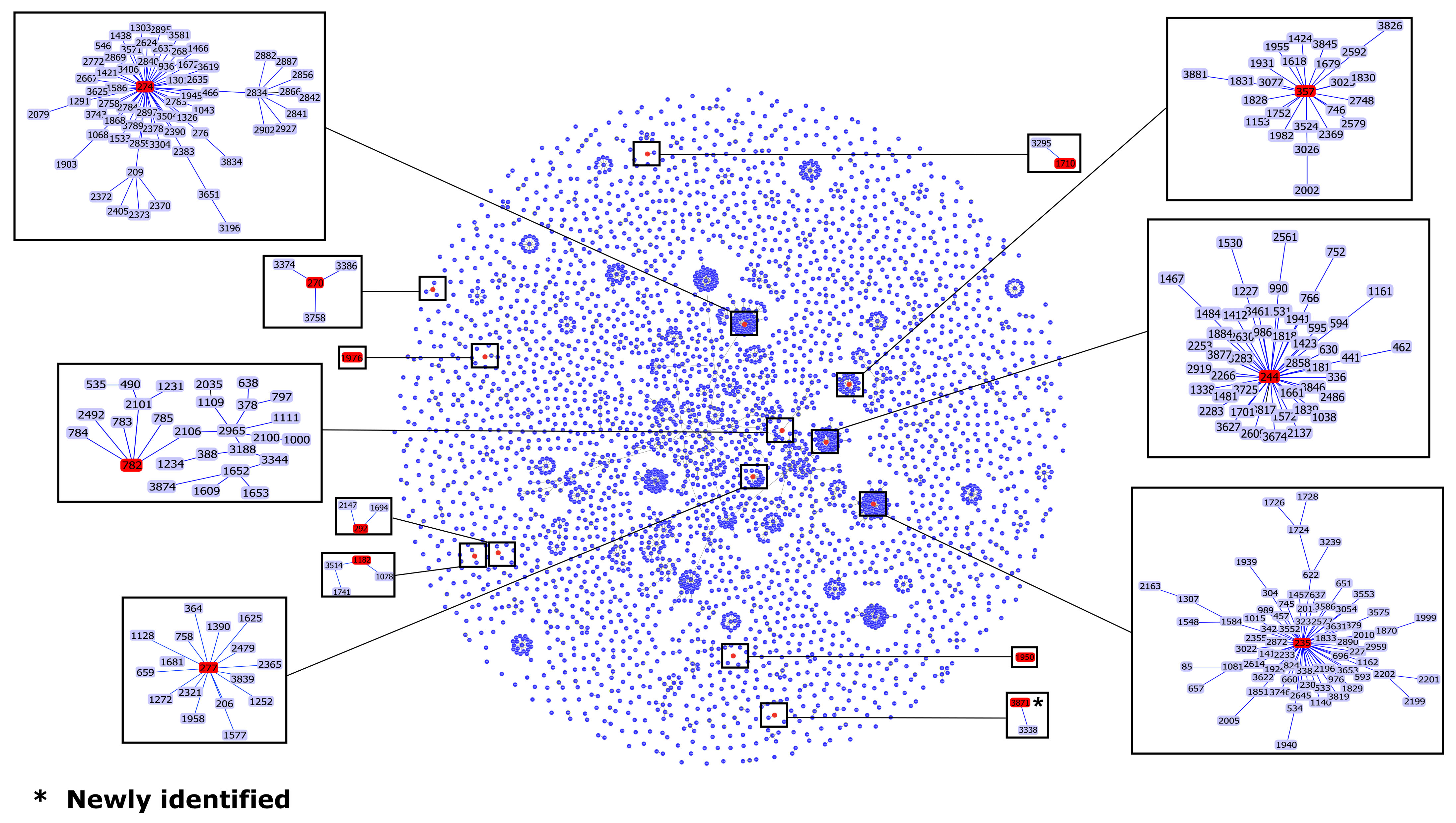

Supplement: Supplementary file 1 — Supplementary Material 1: Figure S1. A minimum spanning tree of MLST among P. aeruginosa isolates. This analysis is displayed by the goeBURST diagram. The MLST database (http://pubmlst.org/paeruginosa, last accessed May 5th, 2022) contained 8251 isolates producing a total of 3912 sequence types (STs). A cluster of linked isolates corresponds to a CC (clonal complex). 2635 STs are divided into 492 CCs and 1277 STs The red boxes show the STs of the 48 isolates in this study. Blue rots are the others in the P. aeruginosa MLST database. [file 12941_2023_600_MOESM1_ESM.jpg]

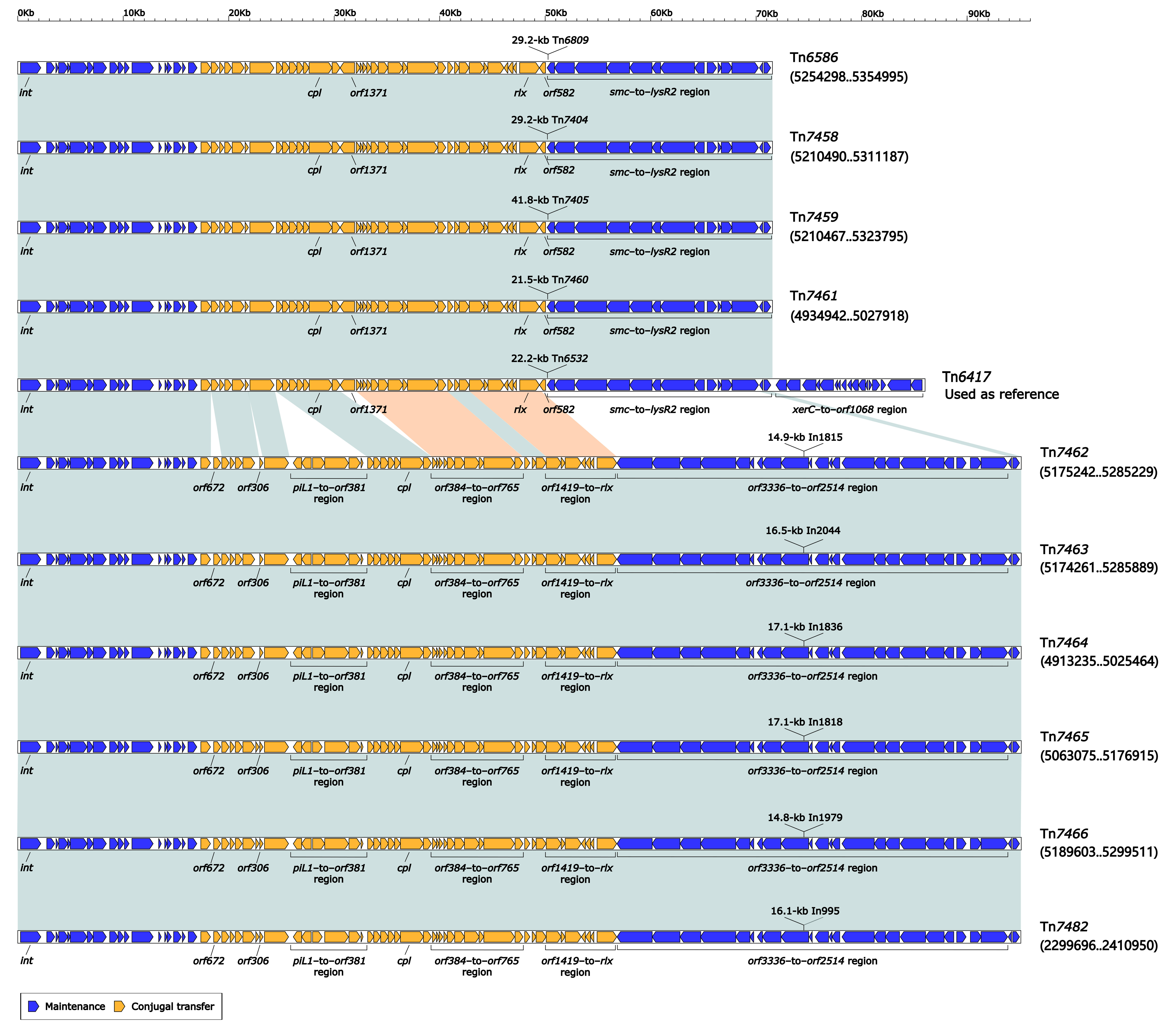

Supplement: Supplementary file 3 — Supplementary Material 3: Figure S3. Comparison of 11 Tn6417->related ICEs. Genes are denoted by arrows. Genes, AGEs, and other features are colored based on their functional classification. Shading in light blue denotes regions of homology (nucleotide identity ≥ 95%), light orange (nucleotide identity < 90%). The accession number of Tn6417 used as reference is EU696790. [file 12941_2023_600_MOESM3_ESM.jpg]

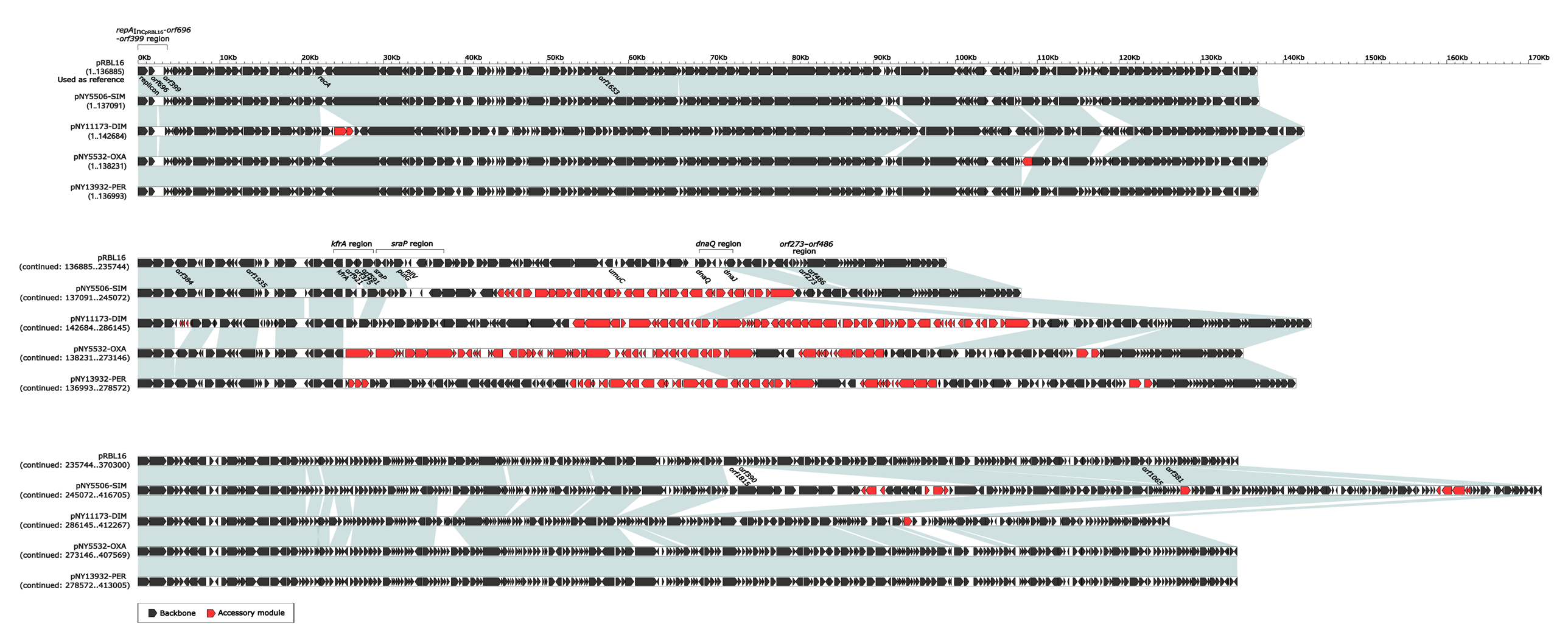

Supplement: Supplementary file 4 — Supplementary Material 4: Figure S4. Comparison of five IncpRBL16 plasmids pRBL16, pNY5506-SIM, pNY11173-DIM, pNY5532-OXA, and pNY13932-PER. Genes are denoted by arrows. Genes, AGEs, and other features are colored based on their functional classification. Shading denotes regions of homology (nucleotide identity ≥ 95%). The accession number of pRBL16 used as reference is CP015879. [file 12941_2023_600_MOESM4_ESM.jpg]

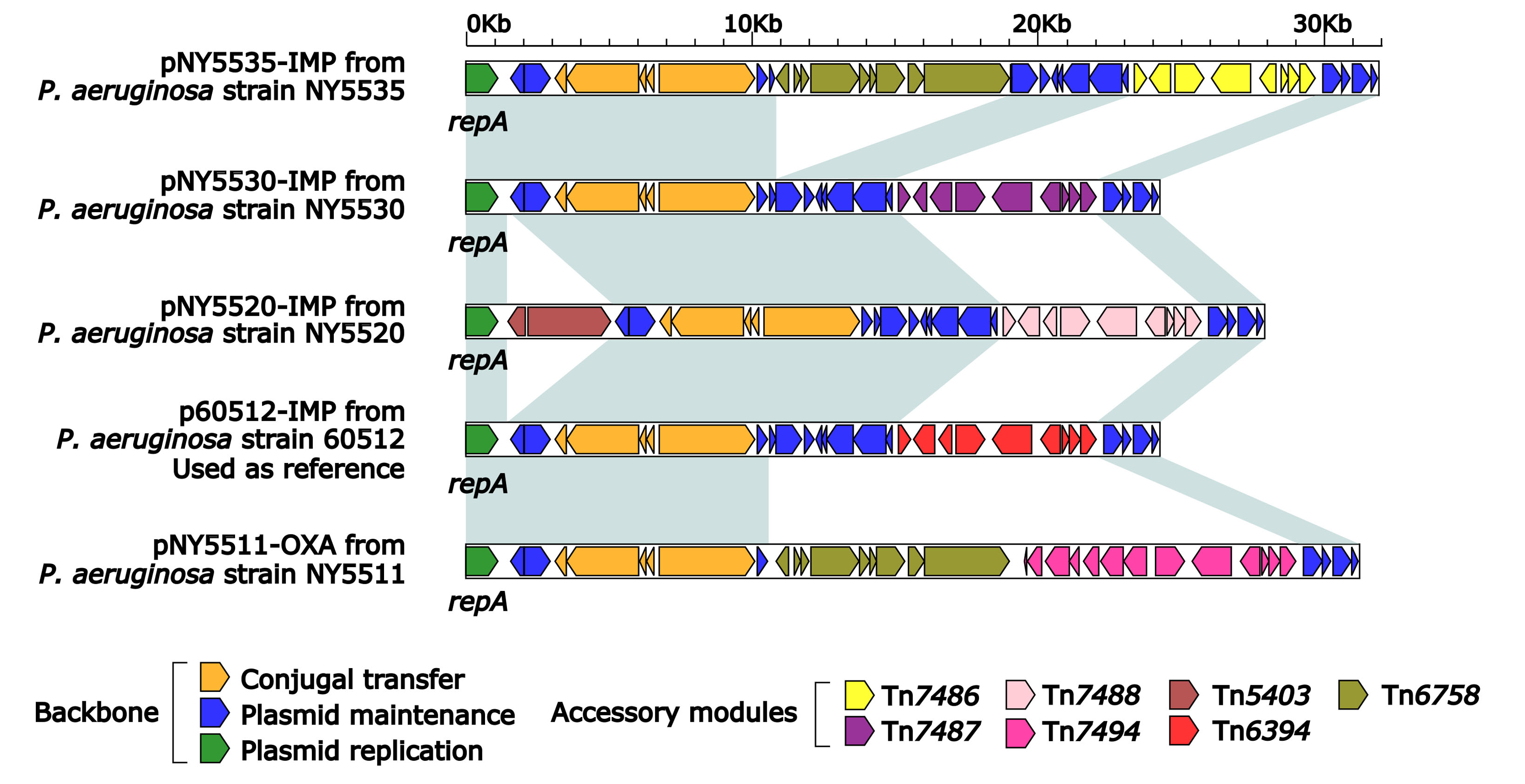

Supplement: Supplementary file 5 — Supplementary Material 5: Figure S5. Comparison of five Incp60512−IMP plasmids p60512-IMP, pNY5535-IMP, pNY5530-IMP, pNY5520-IMP, and pNY5511-OXA. Genes are denoted by arrows. Genes, AGEs, and other features are colored based on their functional classification. Shading denotes regions of homology (nucleotide identity ≥ 95%). The accession number of p60512-IMP used as reference is MF344578. [file 12941_2023_600_MOESM5_ESM.jpg]

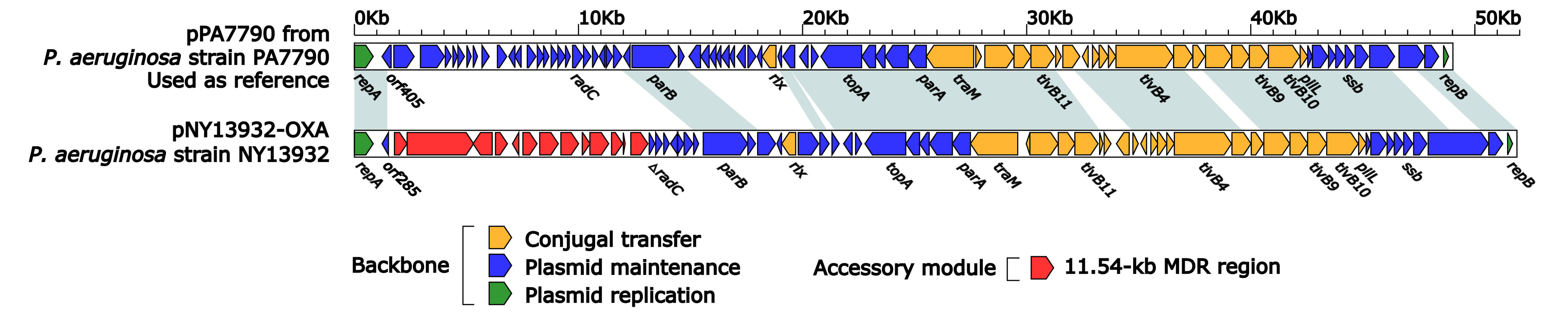

Supplement: Supplementary file 6 — Supplementary Material 6: Figure S6. Comparison of two IncpPA7790 plasmids pPA7790 and pNY13932-OXA. Genes are denoted by arrows. Genes, AGEs, and other features are colored based on their functional classification. Shading denotes regions of homology (nucleotide identity ≥ 95%). The accession number of pPA7790 used as reference is CP015000. [file 12941_2023_600_MOESM6_ESM.jpg]
