# Supplementary material for: Abundant diversity of accessory genetic elements and associated antimicrobial resistance genes in pseudomonas aeruginosa isolates from a single Chinese hospital
Source: Ann Clin Microbiol Antimicrob. 2023 Jun 29;22:51. doi: 10.1186/s12941-023-00600-3 (PMC10311859; doi:10.1186/s12941-023-00600-3)
Supplement: Supplementary file 8 — Supplementary Material 8 [file 12941_2023_600_MOESM8_ESM.docx]

**T****able S2. Sub-regions and resistance genes in the 30 AGEs characterized in this study**

| **Group** | **AGEs** | **Sub-region harbored** | **Resistance marker** | **Resistance phenotype** | **Nucleotide position** |
| --- | --- | --- | --- | --- | --- |
| Tn*6417*-related ICEs | Tn*6417* | Tn*6532* | *aadB* | Aminoglycoside resistance | 5423757..5424290 |
|  |  |  | *qacED1* | Quaternary ammonium compound resistance | 5424447..5424794 |
|  |  |  | *sul1* | Sulphonamide resistance | 5424788..5425627 |
|  |  |  | *mer* locus | Mercuric resistance | 5433944..5437461 |
|  | Tn*6586* | Tn*6809* | *bla*_GES-15_ | β-lactam resistance | 5314565..5315428 |
|  |  |  | *aacA4'* | Aminoglycoside resistance | 5315567..5316121 |
|  |  |  | *aphA15* | Aminoglycoside resistance | 5316454..5317248 |
|  |  |  | *qacED1* | Quaternary ammonium compound resistance | 5318838..5319185 |
|  |  |  | *Δsul1* | Sulphonamide resistance | 5319181..5319707 |
|  |  |  | *cmlA9* | Phenicol resistance | 53203675..5321581 |
|  |  |  | *tetA*(G) | Tetracycline resistance | 5322518..5323693 |
|  |  |  | *mer* locus | Mercuric resistance | 5330106..5333623 |
|  | Tn*7458* | Tn*7404* | *bla*_GES-5_ | β-lactam resistance | 5270757..5271620 |
|  |  |  | *aacA4'* | Aminoglycoside resistance | 5271759..5272313 |
|  |  |  | *aphA15* | Aminoglycoside resistance | 5272646..5273440 |
|  |  |  | *qacED1* | Quaternary ammonium compound resistance | 5275030..5275377 |
|  |  |  | *Δsul1* | Sulphonamide resistance | 5275373..5275899 |
|  |  |  | *cmlA9* | Phenicol resistance | 5276559..5277773 |
|  |  |  | *tetA*(G) | Tetracycline resistance | 5278710..5279885 |
|  |  |  | *mer* locus | Mercuric resistance | 5286298.. 5289815 |
|  | Tn*7459* | Tn*7405* | *bla*_GES-5_ | β-lactam resistance | 5270734..5271597 |
|  |  |  | *aacA4'* | Aminoglycoside resistance | 5271736..5272290 |
|  |  |  | *aphA15* | Aminoglycoside resistance | 5272623..5273417 |
|  |  |  | *qacED1* | Quaternary ammonium compound resistance | 5275007..5275354 |
|  |  |  | Δ*sul1* | Sulphonamide resistance | 5275348..5275876 |
|  |  |  | *cmlA9* | Phenicol resistance | 5276536..5277750 |
|  |  |  | *tetA*(G) | Tetracycline resistance | 5278687..5279861 |
|  |  |  | Δ*bla*_GES-1_ | β-lactam resistance | 5298906..5299142 |
|  |  |  | *mer* locus | Mercuric resistance | 5301989..5302423 |
|  | Tn*7461* | Tn*7460* | *dfrA16* | Trimethoprim resistance | 4993594..4994067 |
|  |  |  | *aadA2* | Aminoglycoside resistance | 4994184..4994963 |
|  |  |  | *qacED1* | Quaternary ammonium compound resistance | 4995127..4995474 |
|  |  |  | *sul1* | Sulphonamide resistance | 4995468..4996307 |
|  |  |  | *erm42* | Macrolide resistance | 4997540..4998451 |
|  |  |  | *strB* | Aminoglycoside resistance | 4998781..4999617 |
|  |  |  | *strA* | Aminoglycoside resistance | 4999617..5000420 |
|  |  |  | *mer* locus | Mercuric resistance | 5003029..5006546 |
|  | Tn*7462* | In1815 | *bla*_OXA-10_ | β-lactam resistance | 5251386..5252186 |
|  |  |  | *aadA1e* | Aminoglycoside resistance | 5252203..5252994 |
|  |  |  | *qacED1* | Quaternary ammonium compound resistance | 5253158..5253505 |
|  |  |  | *sul1* | Sulphonamide resistance | 5253499..5254338 |
|  |  |  | *mer* locus | Mercuric resistance | 5260247..5263767 |
|  | Tn*7463* | In2144 | *bla*_OXA-10_ | β-lactam resistance | 5250405..5251205 |
|  |  |  | *aadA1e* | Aminoglycoside resistance | 5251222..5252013 |
|  |  |  | *qnrVC1* | Fluoroquinolone resistance | 5252246..5252941 |
|  |  |  | *aacA4'* | Aminoglycoside resistance | 5253105..5253659 |
|  |  |  | *qacED1* | Quaternary ammonium compound resistance | 5253828..5254175 |
|  |  |  | *sul1* | Sulphonamide resistance | 5254169..5255008 |
|  |  |  | *mer* locus | Mercuric resistance | 5260917..5264437 |
|  | Tn*7464* | In1836 | *bla*_OXA-10_ | β-lactam resistance | 4989379..4990179 |
|  |  |  | *aadA1e* | Aminoglycoside resistance | 4990196..4990987 |
|  |  |  | *qnrVC1* | Fluoroquinolone resistance | 4991220..4991915 |
|  |  |  | *aacA4'* | Aminoglycoside resistance | 4992079..4992633 |
|  |  |  | *aadB* | Aminoglycoside resistance | 4992703..4993236 |
|  |  |  | *qacED1* | Quaternary ammonium compound resistance | 4993393..4993740 |
|  |  |  | *sul1* | Sulphonamide resistance | 4993734..4994573 |
|  |  |  | *mer* locus | Mercuric resistance | 5000482..5004002 |
|  | Tn*7465* | In1818 | *aadB* | Aminoglycoside resistance | 5140658..5141191 |
|  |  |  | *bla*_OXA-10_ | β-lactam resistance | 5141421..5142221 |
|  |  |  | *aadA1e* | Aminoglycoside resistance | 5142238..5143029 |
|  |  |  | *qnrVC1* | Fluoroquinolone resistance | 5143262..5143957 |
|  |  |  | *aacA4'* | Aminoglycoside resistance | 5144121..5144675 |
|  |  |  | *qacED1* | Quaternary ammonium compound resistance | 5144844..5145191 |
|  |  |  | *sul1* | Sulphonamide resistance | 5145185..5146024 |
|  |  |  | *mer* locus | Mercuric resistance | 5151933..5155453 |
|  | Tn*7466* | In1979 | *aadB* | Aminoglycoside resistance | 5265575..5266108 |
|  |  |  | *aadA2* | Aminoglycoside resistance | 5266497..5267276 |
|  |  |  | *qacED1* | Quaternary ammonium compound resistance | 5267440..5267787 |
|  |  |  | *sul1* | Sulphonamide resistance | 5267781..5268620 |
|  |  |  | *mer* locus | Mercuric resistance | 5274529..5278049 |
|  | Tn*7482* | In995 | *mer* locus | Mercuric resistance | 2321158..2324678 |
|  |  |  | *bla*_IMP-10_ | β-lactam resistance | 2334231..2334971 |
| Tn*1403*-related  transposons or derivatives | Tn*1403* | In28 | *bla*_CARB-2_ | β-lactam resistance | 5158..6024 |
|  |  |  | *cmlA1d* | Phenicol resistance | 6339..7598 |
|  |  |  | *aadA1a* | Aminoglycoside resistance | 7691..8482 |
|  |  |  | ∆*qacED1* | Quaternary ammonium compound resistance | 8646..8970 |
|  |  | Tn*5393c* | *strA* | Aminoglycoside resistance | 15594..16397 |
|  |  |  | *strB* | Aminoglycoside resistance | 16397..17233 |
|  | Tn*7483* | In1791 | *aacA3* | Aminoglycoside resistance | 3353329..3353883 |
|  |  |  | *aadA13* | Aminoglycoside resistance | 3353942..3354739 |
|  |  |  | *bla*_OXA-246_ | β-lactam resistance | 3354968..3355768 |
|  |  |  | *qacED1* | Quaternary ammonium compound resistance | 3355976..3356323 |
|  |  |  | *sul1* | Sulphonamide resistance | 3356317..3357156 |
|  |  |  | *bla*_PER-1_ | β-lactam resistance | 3359425..3360351 |
|  |  |  | *aacC2* | Aminoglycoside resistance | 3370795..3371655 |
|  |  |  | *ΔtmrB* | Tunicamycin resistance | 3371668..3371977 |
|  |  |  | *rmtB* | Aminoglycoside resistance | 3375021..3375776 |
|  |  |  | *bla*_TEM-1_ | β-lactam resistance | 3375946..3376806 |
|  |  | Tn*5393c* | *strA* | Aminoglycoside resistance | 3385530..3386333 |
|  |  |  | *strB* | Aminoglycoside resistance | 3386333..3387169 |
|  | Tn*6846* | In1079 | *aacA4-12* | Aminoglycoside resistance | 6306050..6306604 |
|  |  |  | *bla*_OXA-101_ | β-lactam resistance | 6306685..6307485 |
|  |  |  | *aadA5* | Aminoglycoside resistance | 6307547..6308335 |
|  |  |  | *qacED1* | Quaternary ammonium compound resistance | 6308503..6308850 |
|  |  |  | *sul1* | Sulphonamide resistance | 6308844..6309683 |
|  |  |  | *bla*_PER-1_ | β-lactam resistance | 6311952..6312878 |
|  |  | Tn*5393c* | *strA* | Aminoglycoside resistance | 6327248..6328051 |
|  |  |  | *strB* | Aminoglycoside resistance | 6328051..6328887 |
|  | Tn*7484* | In458 | *aadB* | Aminoglycoside resistance | 1310895..1311428 |
|  |  |  | *aacA3* | Aminoglycoside resistance | 1311502..1312056 |
|  |  |  | *bla*_CARB-2_ | β-lactam resistance | 1312174..1313040 |
|  |  |  | *qacED1* | Quaternary ammonium compound resistance | 1313257..1313604 |
|  |  |  | ∆*sul1* | Sulphonamide resistance | 1313598..1313975 |
|  |  | ∆Tn*5393c* | *strA* | Aminoglycoside resistance | 1318441..1319244 |
|  |  |  | *strB* | Aminoglycoside resistance | 1319244..1320080 |
|  | Tn*7485* | In51 | *aadA6* | Aminoglycoside resistance | 2496163..2497008 |
|  |  |  | *qacED1* | Quaternary ammonium compound resistance | 2497445..2497792 |
|  |  |  | *sul1* | Sulphonamide resistance | 2497786..2498625 |
|  | T1403RE_cNY5525_ | IS*CR3*–t*etA*(G)–*cmlA9* unit | *cmlA9* | Phenicol resistance | 3166375..3167589 |
|  |  |  | *tetA*(G) | Tetracycline resistance | 3168526..3169701 |
|  |  | In1829 | *bla*_OXA-2_ | β-lactam resistance | 3173175..3174002 |
|  |  |  | *aacA4'* | Aminoglycoside resistance | 3174054..3174608 |
|  |  |  | *bla*_OXA-2_ | β-lactam resistance | 3174951..3175778 |
|  |  |  | *qacED1* | Quaternary ammonium compound resistance | 3175914..3176261 |
|  |  |  | *sul1* | Sulphonamide resistance | 3176255..3177094 |
|  | T1403RE_cNY5532_ | In44 | *aacA3* | Aminoglycoside resistance | 5941177..5941731 |
|  |  | IS*CR3*–t*etA*(G)–*cmlA9* unit | *cmlA9* | Phenicol resistance | 5942450..5943664 |
|  |  |  | *tetA*(G) | Tetracycline resistance | 5944601..5945776 |
|  |  | In167 | *bla*_CARB-2_ | β-lactam resistance | 5949298..5950164 |
|  |  |  | *qacED1* | Quaternary ammonium compound resistance | 5950381..5950728 |
|  |  |  | *sul1* | Sulphonamide resistance | 5950722..5951561 |
| Inc_pRBL16_  plasmids | pRBL16 | Plasmid backbone | *ter* locus | Tellurium resistance | 124725..147607 |
|  | pNY5506-SIM | Plasmid backbone | *ter* locus | Tellurium resistance | 124929..147813 |
|  |  | T1403RE_pNY5506-SIM_ | Δ*cmlA1D17* | Chloramphenicol resistance | 181882..182488 |
|  |  |  | *aacA3* | Aminoglycoside resistance | 182537..183091 |
|  |  |  | *qnrVC1* | Fluoroquinolone resistance | 183256..183951 |
|  |  |  | *bla*_VIM-2_ | β-lactam resistance | 190356..191156 |
|  |  |  | *aphA7* | Aminoglycoside resistance | 192990..193805 |
|  |  |  | *mph*(E) | Mecrolide resistance | 196549..197433 |
|  |  |  | *msr*(E) | Mecrolide resistance | 197489..198964 |
|  |  |  | *armA* | Aminoglycoside resistance | 201263..202036 |
|  |  |  | *sul1* | Sulphonamide resistance | 206484..207323 |
|  |  |  | *qacED1* | Quaternary ammonium compound resistance | 207317..207664 |
|  |  |  | *catB3q* | Chloramphenicol resistance | 209203..209835 |
|  |  |  | *ereA1c* | Erythromyci resistance | 209945..211165 |
|  |  |  | *bla*_SIM-1_ | β-lactam resistance | 211661..212401 |
|  | pNY11173-DIM | Plasmid backbone | *ter* locus | Tellurium resistance | 130083..147409  149171..153970 |
|  |  | Tn*6562* | *aadA1a* | Aminoglycoside resistance | 207593..208384 |
|  |  | Tn*6488* | *bla*_OXA-4_ | β-lactam resistance | 208497..209327 |
|  |  | T1403RE_pNY11173-DIM_ | *catB3* | Chloramphenicol resistance | 209446..210078 |
|  |  |  | *dfrB10* | Trimethoprim resistance | 210344..210580 |
|  |  |  | *bla*_DIM-2_ | β-lactam resistance | 210731..211486 |
|  |  |  | *strB* | Streptomycin resistance | 220136..220972 |
|  |  |  | *strA* | Streptomycin resistance | 220972..221775 |
|  |  |  | *aacA3* | Aminoglycoside resistance | 232002..232556 |
|  |  |  | *catB11c* | Chloramphenicol resistance | 234024..234656 |
|  |  |  | *qacED1* | Quaternary ammonium compound resistance | 235298..235645 |
|  |  |  | Δ*sul1* | Sulphonamide resistance | 235639..236610 |
|  |  |  | Δ*cmlA9* | Chloramphenicol resistance | 236827..237855 |
|  |  |  | *sul1* | Sulphonamide resistance | 239979..240818 |
|  |  |  | *qacED1* | Quaternary ammonium compound resistance | 240812..241159 |
|  |  |  | *dfrA1z* | Trimethoprim resistance | 241712..242185 |
|  |  |  | *catB11c* | Chloramphenicol resistance | 242993..243625 |
|  |  |  | *aacA3* | Aminoglycoside resistance | 245096..245650 |
|  |  |  | *qnrVC6* | Fluoroquinolone resistance | 245815..246471 |
|  | pNY5532-OXA | Plasmid backbone | *ter* locus | Tellurium resistance | 125959..148843 |
|  |  | T1403RE_pNY5532-OXA_ | *aacC2* | Aminoglycoside resistance | 197457..198317 |
|  |  |  | *sul1* | Sulphonamide resistance | 202976..203815 |
|  |  |  | *qacED1* | Quaternary ammonium compound resistance | 203809..204156 |
|  |  |  | *bla*_OXA-246_ | β-lactam resistance | 204364..205164 |
|  |  |  | *cmlA1k* | Chloramphenicol resistance | 205268..206527 |
|  |  |  | *aadA16* | Aminoglycoside resistance | 206782..207579 |
|  |  |  | *aacA3* | Aminoglycoside resistance | 207638..208192 |
|  | pNY13932-PER | Plasmid backbone | *ter* locus | Tellurium resistance | 124831..147715 |
|  |  |  | *strA* | Streptomycin resistance | 163569..164372 |
|  |  |  | *strB* | Streptomycin resistance | 164372..165208 |
|  |  | T1403RE_pNY13932-PER_ | *sul1* | Sulphonamide resistance | 191525..192364 |
|  |  |  | Δ*qacED1* | Quaternary ammonium compound resistance | 192358..192642 |
|  |  |  | *bla*_PER-1_ | β-lactam resistance | 197310..198236 |
|  |  |  | *sul1* | Sulphonamide resistance | 200505..201344 |
|  |  |  | Δ*qacED1* | Quaternary ammonium compound resistance | 201338..201622 |
|  |  |  | *bla*_PER-1_ | β-lactam resistance | 206290..207216 |
|  |  |  | *sul1* | Sulphonamide resistance | 209485..210324 |
|  |  |  | *qacED1* | Quaternary ammonium compound resistance | 210318..210665 |
|  |  |  | *bla*_OXA-246_ | β-lactam resistance | 210873..211673 |
|  |  |  | *cmlA1k* | Chloramphenicol resistance | 211777..213036 |
|  |  |  | *aadA13* | Aminoglycoside resistance | 213291..214088 |
|  |  |  | *aacA3* | Aminoglycoside resistance | 214147..214701 |
| Inc_p60512-IMP_  plasmids | p60512-IMP | Tn*6394* | *bla*_IMP-1_ | β-lactam resistance | 15678..16418 |
|  |  |  | *aacA7* | Aminoglycoside resistance | 16557..17015 |
|  | pNY5535-IMP | Tn*7486* | *bla*_IMP-10_ | β-lactam resistance | 23938..24678 |
|  | pNY5530-IMP | Tn*7487* | *aacA7* | Aminoglycoside resistance | 15676..16134 |
|  |  |  | *bla*_IMP-10_ | β-lactam resistance | 16268..17008 |
|  | pNY5520-IMP | Tn*7488* | *bla*_IMP-10_ | β-lactam resistance | 19347..20087 |
|  |  |  | *aacA7* | Aminoglycoside resistance | 20226..20684 |
|  | pNY5511-OXA | Tn*6758* | *mer* locus | Mercuric resistance | 10865..14383 |
|  |  | Tn*7494* | *sul1* | Sulphonamide resistance | 20292..21131 |
|  |  |  | *qacED1* | Quaternary ammonium compound resistance | 21125..21472 |
|  |  |  | *aadB* | Aminoglycoside resistance | 21629..22162 |
|  |  |  | *aadA1* | Aminoglycoside resistance | 22227..23018 |
|  |  |  | *bla*_OXA-10_ | β-lactam resistance | 23035..23835 |
| Inc_pPA7790_  plasmid | pNY13932-OXA | 11.54-kb MDR region | *aadB* | Aminoglycoside resistance | 6296..6829 |
|  |  |  | *catB3* | Aminoglycoside resistance | 7519..8151 |
|  |  |  | *bla*_OXA-1_ | β-lactam resistance | 8270..9100 |
|  |  |  | *aadA1a* | Aminoglycoside resistance | 9213..10004 |
|  |  |  | *qacED1* | Quaternary ammonium compound resistance | 10168..10515 |
|  |  |  | *sul1* | Sulphonamide resistance | 10509..11348 |
